# Sitting and standing intention can be decoded from scalp EEG recorded prior to movement execution

**DOI:** 10.3389/fnins.2014.00376

**Published:** 2014-11-25

**Authors:** Thomas C. Bulea, Saurabh Prasad, Atilla Kilicarslan, Jose L. Contreras-Vidal

**Affiliations:** ^1^Functional and Applied Biomechanics Section, Rehabilitation Medicine Department, National Institutes of HealthBethesda, MD, USA; ^2^Laboratory for Non-invasive Brain-Machine Interface Systems, Department of Electrical and Computer Engineering, University of HoustonHouston, TX, USA

**Keywords:** EEG, electroencephalography, movement-related cortical potentials, classification, brain-machine interface, mobile neuroimaging, lower extremity

## Abstract

Low frequency signals recorded from non-invasive electroencephalography (EEG), in particular movement-related cortical potentials (MRPs), are associated with preparation and execution of movement and thus present a target for use in brain-machine interfaces. We investigated the ability to decode movement intent from delta-band (0.1–4 Hz) EEG recorded immediately before movement execution in healthy volunteers. We used data from epochs starting 1.5 s before movement onset to classify future movements into one of three classes: stand-up, sit-down, or quiet. We assessed classification accuracy in both externally triggered and self-paced paradigms. Movement onset was determined from electromyography (EMG) recordings synchronized with EEG signals. We employed an artifact subspace reconstruction (ASR) algorithm to eliminate high amplitude noise before building our time-embedded EEG features. We applied local Fisher's discriminant analysis to reduce the dimensionality of our spatio-temporal features and subsequently used a Gaussian mixture model classifier for our three class problem. Our results demonstrate significantly better than chance classification accuracy (chance level = 33.3%) for the self-initiated (78.0 ± 2.6%) and triggered (74.7 ± 5.7%) paradigms. Surprisingly, we found no significant difference in classification accuracy between the self-paced and cued paradigms when using the full set of non-peripheral electrodes. However, accuracy was significantly increased for self-paced movements when only electrodes over the primary motor area were used. Overall, this study demonstrates that delta-band EEG recorded immediately before movement carries discriminative information regarding movement type. Our results suggest that EEG-based classifiers could improve lower-limb neuroprostheses and neurorehabilitation techniques by providing earlier detection of movement intent, which could be used in robot-assisted strategies for motor training and recovery of function.

## Introduction

Robot-assisted therapies have shown promising results, compared to traditional therapy, for functional recovery of movement after injury in the upper and lower extremities (Winchester et al., 2005; Hogan and Krebs, 2011). These neurorehabilitation paradigms could be improved by faster and more robust detection of movement intent where it originates in the brain. Incorporation of a brain machine interface (BMI) can reduce the latency between motor planning in the cortex and activation of a device to execute (or assist) the movement, thereby enhancing the opportunity for brain plasticity and motor recovery (Daly and Wolpaw, 2008). The intuitive nature of a BMI based on signals directly related to intended movement could be advantageous for rehabilitation by expediting adaptation of the brain to the BMI algorithm and the robotic device. Electroencephalography (EEG) provides a non-invasive method for imaging brain activity with enough time resolution to exert control over an assistive device. Many strategies for deploying EEG in a BMI by detecting movement intent (imagined and real) have been reported (Pfurtscheller et al., 1996, 2006; Wolpaw et al., 2002; Millán et al., 2004; Qin et al., 2004; Hung et al., 2005; Morash et al., 2008). These systems typically leverage one of two phenomena to detect movement intent: event related (de)synchronization (ERD/ERS) and movement related slow cortical potentials (MRPs). ERD, a decrease of power in alpha and beta bands, is typically localized to the contralateral sensorimotor areas before movement while ERS, a power increase, has been observed after movement (Pfurtscheller and Lopes da Silva, 1999). Modulation of these sensorimotor rhythms has been employed for classification of imagined (Pfurtscheller et al., 2006; Pfurtscheller and Neuper, 2006) and executed (Morash et al., 2008) movements with some success. ERD has also shown capacity to categorize gross lower extremity tasks, including differentiation of right and left leg motor imagery (Boord et al., 2010) and identification of imagined standing (Zhong et al., 2007).

MRPs are slow negative potentials observed in EEG preceding movement. MRPs can be divided into two segments: the first begins as early as 2 s before movement onset and has been observed over the entire pre-supplementary motor area (SMA), and over the SMA and lateral premotor cortex according to somatotopic organization (Ikeda et al., 1992; Hallett, 1993; Shibasaki and Hallett, 2006; Bai et al., 2011). The second, or late, segment typically has a steeper negative slope and is observed in the contralateral primary motor cortex (M1) and lateral premotor cortex according to precise somatotopic arrangement. These potentials are well established in upper and lower extremity movements both real and imagined (Boschert and Deecke, 1986; Shibasaki and Hallett, 2006). Interestingly, MRPs recorded from EEG preceding toe, foot, and ankle movements tend to be larger on the ipsilateral side of the brain, which is the opposite of upper extremity movements that create larger MRPs on the contralateral side (Brunia and Van Den Bosch, 1984; Boschert and Deecke, 1986). This paradoxical lateralization of the MRP during foot movements may be explained by its localization along the midline deep within the precentral gyrus of the motor cortex, thereby directing electrical current from activation of these cell columns to the opposite hemisphere.

The type and sequence of movement affects MRPs recorded from EEG. MRPs appear to be more pronounced during self-initiated movements compared to triggered movements (Jahanshahi et al., 1995; Cui and MacKinnon, 2009); the difference appears to be further enhanced if the timing of the triggered movements is variable (Jankelowitz and Colebatch, 2002). In the case of finger movements, force level (Slobounov et al., 2002), finger sequence (Bortoletto et al., 2011), and task complexity (Shibasaki and Hallett, 2006) all appear to modulate the MRP. MRP amplitude was found to be highly correlated to joint torque and electromyography (EMG) amplitude during isolated elbow flexion (Siemionow et al., 2000). In the lower extremity, the rate of torque development appears to influence the late MRPs preceding isolated ankle movements (do Nascimento et al., 2006). Slow negative shifts in EEG similar to MRPs have been observed during coordinated movements of the lower extremity, including rising onto the toes (Saito et al., 1996) and self-paced forward postural sway (Slobounov et al., 2005). The direction of gait initiation and stepping has been reported to influence both the slope and magnitude of MRPs (do Nascimento et al., 2005). These previously published studies suggest that slow developing, movement related potentials observed prior to movement may contain discriminative information regarding the movement that is being performed. Further, MRPs appear to provide an appropriate measure for timing of afferent feedback to induce long term potentiation of cortical projections. As demonstrated in the tibialis anterior muscle, only peripheral stimulation delivered at the peak of the MRP increased motor evoked potentials from transcranial magnetic stimulation (TMS) targeting the ankle area of the motor cortex (Mrachacz-Kersting et al., 2012).

Because of their small amplitude and low frequency content, the best way to extract MRPs from EEG recording is to average across many trials of the same movement. Single trial classification of movement intention from MRPs is possible, but achieving high accuracy can be difficult. Classification typically involves several steps, including signal pre-processing, feature extraction, dimensionality reduction, and finally feature classification (Bashashati et al., 2007). Numerous approaches to these steps have resulted in application of many machine learning, feature selection, and pattern recognition techniques for classification of movement intent and direction based on EEG signals (Garrett et al., 2003; Peterson et al., 2005; Bai et al., 2007; Lotte et al., 2007). The first example of a BMI-based spelling device utilized slow cortical potentials derived from a motor imagery task to provide individuals with amyotrophic lateral sclerosis control of a cursor on a screen (Birbaumer et al., 1999). Two individuals were able to achieve accuracies greater than 75% after 327 and 288 training sessions. Recent studies have demonstrated success in utilizing MRPs extracted via low frequency or delta band EEG, including classification of finger movement (Liao et al., 2007), joystick direction (Waldert et al., 2008), wrist movement direction (Vuckovic and Sepulveda, 2008), direction of a center out reaching task (Robinson et al., 2013), and movement intention in a self-paced reaching task (Lew et al., 2012). The latter study showed higher detection accuracy using the lower delta band than alpha (7–13 Hz) or beta (13–20 Hz) bands. MRPs have also been successfully deployed for classification of lower extremity movements. At the ankle, MRPs have been used to detect movement intention in healthy subjects with average accuracy of 82.5% for movement execution, and with slightly lower accuracy for motor imagery (64.5%) and attempted movement in stroke patients (55%) (Niazi et al., 2011). Similar accuracies were reported in a study that did not incorporate an individual-specific training phase (Niazi et al., 2013), further supporting the robustness of MRP as a BMI target. In addition to movement intention, MRPs recorded during imagined plantar flexion have also been used to distinguish between two different rates of torque development (do Nascimento and Farina, 2008). Recent studies have demonstrated that MRPs recorded from EEG can be deployed in real-time BMIs. In one, MRPs preceding imagined ankle dorsiflexion were identified online to trigger electrical stimulation of the tibialis anterior (Niazi et al., 2012). Not only did this study show feasibility of MRPs for use in a BMI, but it also demonstrated the potential benefits BMMI-based neurorehabilitation since motor evoked potentials from TMS were enhanced following the intervention in healthy individuals. Another study showed that delta band EEG could reliably ascertain ankle movement initiation in real time with a mean latency of 315 ms (Xu et al., 2014).

In addition to detecting and classifying movement type, sparse networks of low frequency EEG have also been successful in decoding kinematics and EMG activity during various movements, including decoding of hand grasping patterns (Agashe and Contreras-Vidal, 2013), hand and finger velocity (Bradberry et al., 2010; Liu et al., 2011; Paek et al., 2014), and muscle synergies during reaching (Beuchat et al., 2013). Additionally, peri-movement neural activity representative of movement direction has been observed in electrocorticographic (ECoG) signals over primary motor, premotor, posterior-parietal, and lateral prefrontal cortex (Ball et al., 2009). Action intention can also be decoded from fMRI data recorded from a wide cortical network, spanning from the parieto-occiptial sulcus through the prefrontal cortex, both preceding and during movement execution (Gallivan et al., 2011). Taken together these studies suggest non-invasive EEG recorded from large areas of the scalp immediately prior to movement execution could carry useful information about movement.

EEG has been used to examine cortical activity during gait, including studies demonstrating that intra-stride changes in spectral power are coupled to gait cycle (Gwin et al., 2011) and that level of user-involvement in robotic-assisted walking alters gait-related patterns of electrocortical activity (Wagner et al., 2012). Low frequency EEG also appears to carry useful information regarding walking. A recent study showed that features corresponding to frequencies less than 2 Hz were the most heavily weighted during single trial classification of walking and pointing direction (Velu and de Sa, 2013). Delta-band EEG was used to classify walking intention in one individual with paraplegia using a robotic exoskeleton with accuracy greater than 98% (Kilicarslan et al., 2013) and to decode lower limb kinematics during walking in healthy individuals (Presacco et al., 2011, 2012). MRPs have also been used with a matched filtering technique to detect single-trial step initiation (Jiang et al., 2014). An important consideration for application of low frequency EEG to the study of whole-body movements such as walking or sit/stand transition is the presence of movement-related artifacts. A recent study showed similar power spectral density patterns from an accelerometer mounted on the head and from EEG electrodes (Castermans et al., 2014). Interestingly, the patterns were similar only at higher walking speeds, while differences between the accelerometer and EEG were observed at slower speeds. The study did not compare spectral patterns from EEG during walking without the rigid plate and linkage assembly used to mount the accelerometer on the head, so the effect of its mass and inertia remains unknown. Also, the study did not employ active EEG electrodes which provide amplification at the electrode to minimize movement artifacts and increase signal-to-noise ratio. Spatial filtering techniques, such as independent component analysis (Delorme et al., 2007), may be used to isolate gait-related artifact, but the effectiveness of these techniques is still under investigation. In one study, gait-related artifact remained in many independent components of EEG, resulting in development of a template subtraction technique to clean EEG collected during walking (Gwin et al., 2010). This type of template regression would not be appropriate for studying cortical contribution to locomotion because all signals coupled to the gait cycle would likely be removed. Another technique utilizes principal component analysis to compare sliding windows of EEG to a baseline recording, thereby removing high amplitude artifacts (Mullen et al., 2013); this approach may be better suited for removing movement artifacts but has not yet been applied to gait. Thus, the feasibility of utilizing EEG to study cortical activations during whole-body movement tasks is an ongoing area of research. Nevertheless, an inherent advantage of MRPs is their presence in EEG recorded before movement, when motion artifacts are minimized.

In this study we examined the use of non-invasive EEG recorded prior to movement execution to discriminate a user's intent to perform two coordinated whole body movements—rising from a seated to standing posture and lowering from a standing to a seated posture—in a three class problem, where the third class constituted no movement or “quiet”; this class included data collected during quiet standing and quiet sitting. Based on the previous body of evidence regarding the discriminative nature of MRPs with regards to movement, we utilized delta band EEG to build our features for classification. We trained and tested our classifier using time periods before executed movements, as opposed to cue-based imagery, so we could precisely align EEG recordings with movement onset detected from EMG recordings. We studied classification accuracy during two different paradigms: a self-initiated series of stand-to-sit and sit-to-stand transitions and transitions which were cued by an audio trigger. Because triggered movements are reported to produce less prominent MRPs (Jankelowitz and Colebatch, 2002; Cui and MacKinnon, 2009), this protocol allowed us to examine the effect of MRP signal to noise ratio on classification accuracy. We utilized time-embedding and concatenation of EEG channels from the time before movement execution to create a feature vector of high dimension to classify the intended movement (stand-up, sit-down, or quiet). Given the autoregressive nature of EEG signals (Muller et al., 2003) and the underlying neurophysiology (e.g., volume conduction), we assume that the recorded EEG originates from a system with fewer degrees of freedom than our feature vector dimensions, resulting in a manifold data structure. Recent advances in machine learning have resulted in algorithms which preserve the local structure of a manifold data set in a reduced dimensional subspace (Sugiyama, 2007; Li et al., 2012) thereby enhancing the discriminative power of the data set. Based on the observation that information pertinent to movement is contained in low frequency EEG, we hypothesized that applying a locality preserving dimensionality reduction technique to our high dimensional feature vector derived from time-embedded and spatially diverse delta band EEG would reveal its underlying discriminative structure. We coupled this supervised data reduction with a Gaussian mixture model classifier to test if we could reliably ascertain the intended movement of the user from offline analysis of EEG recordings. We believe such a classifier could eventually be deployed in a real-time BMI system to control an assistive device or as a component of a neurorehabilitation paradigm to restore motor control.

## Methods

### Data collection

Ten healthy adults (6 male, 4 female) with no history of neurological disease participated in the study after giving informed consent. This study protocol was approved by the Institutional Review Board at the University of Houston. Participants completed two trials of 10 alternating sit-to-stand and stand-to-sit transitions; one trial was self-paced and one trial was cued via audio trigger. Each trial began with the participant standing quietly in an upright posture for 15 s. In the triggered trial, an audio cue (beep) was given after which point the participant initiated a transition to a seated posture. The seated posture was held for a period ranging randomly from 3 to 10 s, after which a second audio cue was given to initiate the transition from sit-to-stand. The standing posture was held for another (random) 3–10 s interval, at which point the process was repeated until 20 transitions (10 of each) were completed. The procedure for the self-paced trial was similar. After 15 s of quiet standing, the participant was instructed via verbal cue to begin the self-initiated stand-to-sit and sit-to-stand transitions. The participant was instructed to wait for a random interval of 3–10 s before self-initiating the next transition. Finally, the participant was notified by verbal cue once he/she had completed 20 self-initiated transitions.

Time-locked EMG and EEG data were collected simultaneously using a previously developed data collection system (Bulea et al., 2013). Surface EMG (Biometrics, Ltd, Ladysmith, VA) was recorded at 1000 Hz bilaterally from the tibialis anterior, gastrocnemius, biceps femoris, and vastus lateralis. Whole scalp, active electrode, 64-channel EEG (Brain Products, GmbH, Morrisville, NC) were collected at 1000 Hz and labeled by the 10–20 international system. The impedance of each EEG electrode was maintained below 25 kΩ for the entire data collection.

### Data analysis for classification of movement intent

#### Preprocessing

**Figure 1:**
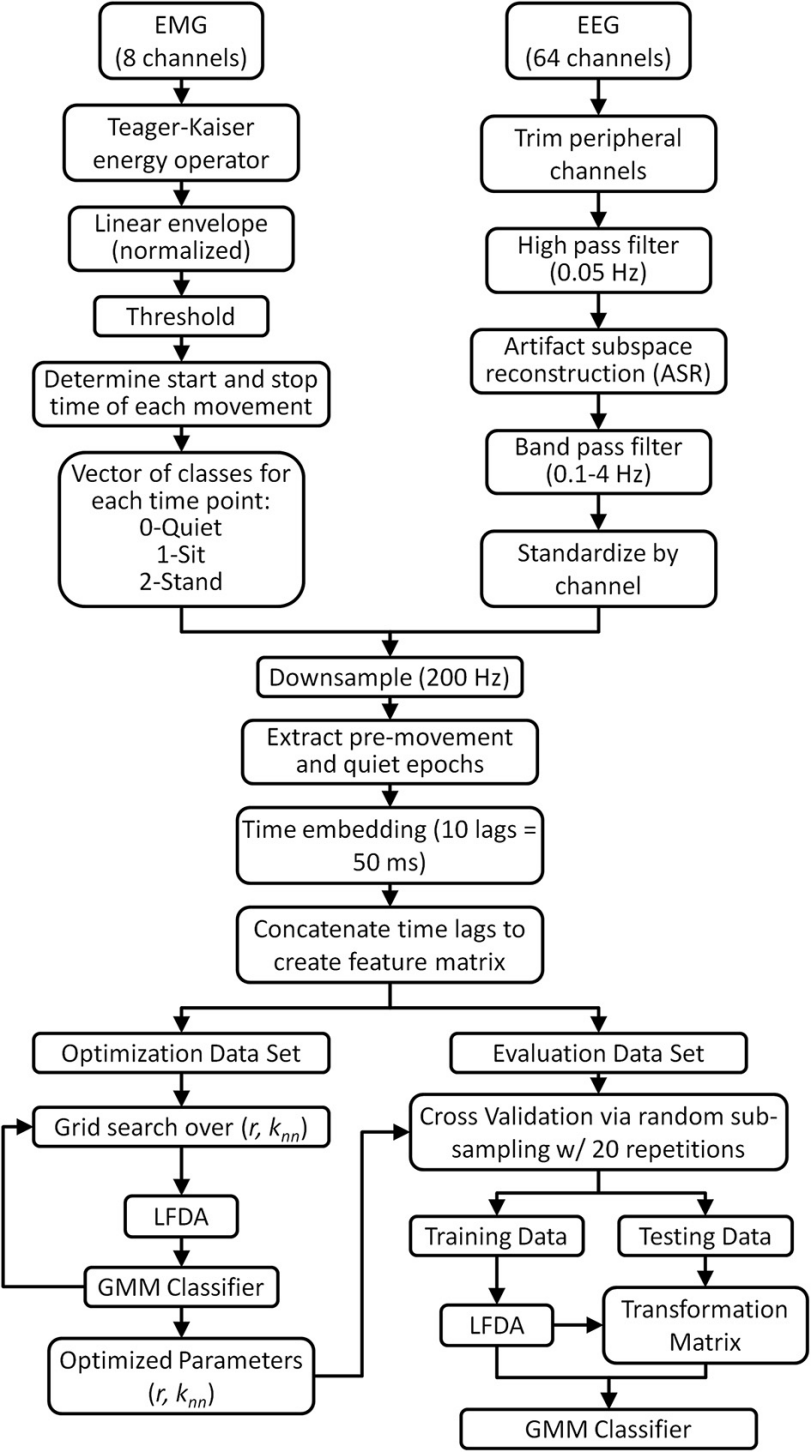
**Flow chart describing the EMG and EEG data processing for neural decoding of sitting and standing movement**. A threshold of 3 standard deviations was applied to the EMG linear envelope to identify quiet periods and periods of movement (sitting and standing). Only pre-movement epochs (1.5 s before movement to movement onset) and quiet epochs (1.5 s after movement completion to 1.5 s before next movement) were retained for analysis. As a control, a separate decoding analysis using movement epochs (movement onset to 1.5 s after onset) was also performed. Artifact subspace reconstruction (ASR) algorithm, available as a plug-in for EEGLAB software (Delorme and Makeig, 2004), was applied to eliminate artifacts from EEG data during pre-processing. Note that the optimization and evaluation data sets are mutually exclusive.

All data analysis and classifier optimization and evaluation were performed off-line using custom software in Matlab (Mathworks, Natick, MA). The data processing and classification methodology is shown in Figure 1. Peripheral EEG channels susceptible to eye blinks and facial/cranial muscle activity were removed from offline analysis (all channels labeled Fp, AF, FT, T, TP, O, PO, and F5-8, P5-8) resulting in 28 channels being retained for classification. EEG signals were then high pass filtered at 0.05 Hz using a zero-phase 8th order Butterworth filter. Next, we removed transient, high-amplitude artifacts from stereotypical (e.g., eye blinks) and non-stereotypical (e.g., movement, muscle bursts) using an automated artifact rejection method termed Artifact Subspace Reconstruction (ASR) (Mullen et al., 2013) which is available as a plug-in for EEGLAB software (Delorme and Makeig, 2004). ASR uses a sliding window technique whereby each window of EEG data is decomposed via principal component analysis so it can be compared statistically with data from a clean baseline EEG recording, collected here as 1 min of EEG recorded during quiet standing. Within each sliding window the ASR algorithm identifies principal subspaces which significantly deviate from the baseline EEG and then reconstructs these subspaces using a mixing matrix computed from the baseline EEG recording. In this study, we used a sliding window of 500 ms and a threshold of 3 standard deviations to identify corrupted subspaces. After ASR, the cleaned EEG was band pass filtered with a zero phase, 3rd order Butterworth filter from 0.1 to 4 Hz to isolate the delta band activity. The EEG data were then standardized by channel by subtracting the mean and dividing by the standard deviation (z-score).

EMG recordings from the lower extremity muscles were used to determine movement onset of each stand-to-sit and sit-to-stand transition. First, the Teager-Kaiser energy operator was applied to each EMG channel to enhance the signal-to-noise ratio for onset detection (Li et al., 2007). Next, each EMG channel was detrended, band pass filtered (15–300 Hz), rectified, and low pass filtered at 3 Hz to compute the linear envelope. Then, the linear envelope of each muscle was thresholded into a binary signal which was equal to 1 when the envelope exceeded its mean baseline value during quiet standing and sitting by more than 3 standard deviations (Hodges and Bui, 1996) and zero when it was within 3 standard deviations of baseline. The baseline period of EMG activity before each movement was identified *a posteriori* by visual inspection starting with the initial 15 s of rest before the first movement. The baseline period between each successive sit-to-stand and stand-to-sit transition comprised at least 2 s. Movement onset for each transition was determined when any of the 8 thresholded EMG envelopes transitioned from rest (0) to active (1). Likewise, the end of each movement was determined when all 8 channels returned to rest (0). The algorithmically determined periods of activity were visually inspected for accuracy. Using prior knowledge of the experimental protocol (i.e., the order of the stand-to-sit and sit-to-stand transitions), the periods of muscle activity were labeled as stand-to-sit or sit-to-stand. Note that for some trials, gastrocnemius muscles were active during the quiet stance phase and/or biceps femoris EMG was contaminated by artifact from the leg during sitting, thereby increasing the standard deviation in these channels and limiting the ability to determine the true state using that muscle. When these periods of activity/artifact were observed visually, these muscles were removed from the trial; in this case the user activity was assessed using the remaining 6 muscles.

Next, the time-locked EEG and EMG data were downsampled to 200 Hz. EEG data were then epoched into pre-movement, post-movement and quiet periods based on the thresholded (binary) EMG signal. Each pre-movement epoch consisted of data from 1.5 s before movement onset up to movement onset. EEG data from 1.5 s after movement completion until 1.5 s before the next movement onset, with a maximum of 5 s, comprised the quiet epochs. These epochs were then concatenated into a single time series containing alternate periods of quiet and pre-movement. For control purposes, we also created a second time series of data containing concatenated quiet epochs and epochs of EEG from movement onset to 1.5 s after movement onset (post-movement epochs).

The concatenated EEG data sets comprised the three-class classification problem for each trial; each time point of the quiet epochs was labeled as class 0 (quiet) while each time point of each pre-movement epoch was labeled according to the type of movement it preceded: class 1 (stand-to-sit) or class 2 (sit-to-stand). Next, a time-embedded feature matrix was constructed for each trial. Each time point in the feature matrix was a vector composed of 10 lags, corresponding to 50 ms in the past, of EEG data. The number of lags and embedded time interval was chosen based on previous studies demonstrating accurate decoding of movement kinematics from low frequency EEG (Bradberry et al., 2010; Presacco et al., 2011). The feature vector for each time point was constructed by concatenating the 11 lags (the current time point plus the 10 prior) for each channel into a single vector of length 11 × *N*, where *N* is the number of EEG channels used for classification (for this study, *N* = 28). To avoid the problem of missing data, the feature matrix was buffered by starting at the 11th EEG sample of each epoch, resulting in a feature matrix of dimension [*M_t_*−*L*] × [11 × *N*] for each trial of self-initiated and triggered movements where *M_t_* is the number of time points in each trial and *L* is the number of past time lags multiplied by the number of epochs in each trial (for this study, *L* = 10^*^41 = 410). On average, there were 18,442 ± 2110 time points in each feature matrix, with exactly 2900 time points for class 1 and 2900 time points for class 2 while the remaining time points represented class 0. For all subjects, the original dimensionality of the feature space was 308 (11 × *N*).

#### Dimensionality reduction

Since our EEG-based feature vectors were of relatively high dimension and were composed of time lagged and spatially distributed samples, we assumed our original dataset to represent a manifold which may contain multimodal within-class distributions. Furthermore, we sought to classify gross motor intention and therefore had a limited number of classes (in this case there were three: quiet, stand-to-sit, and sit-to-stand). Thus, we performed dimensionality reduction on our feature matrices to eliminate any redundant features, reduce computational complexity, prevent over-fitting during classifier training and increase classification performance. Many techniques have been reported for dimensionality reduction in EEG based classifiers, including principal component analysis (PCA), linear discriminant analysis (LDA), and genetic algorithm (GA) (Bashashati et al., 2007; Lotte et al., 2007). Consideration of the task, neurophysiology and EEG recording system suggests that a supervised dimensionality reduction technique could improve feature selection for classification purposes. EEG data generally have a low signal-to-noise ratio and unsupervised linear dimensionality reduction techniques may be affected by these signal distortions. PCA reduces dimensionality by maximizing data variance in the projected subspace via a linear transformation. The transformation, dictated by the eigenvectors that correspond to the largest eigenvalues of the data covariance matrix, is unsupervised and can discard useful information for classification that is contained in the lower energy dimensions of the original data (Prasad and Bruce, 2008). In contrast, LDA is a supervised dimensionality reduction technique since it attempts to maximize between-class scatter while minimizing within-class scatter in the projected subspace. However, LDA has difficulty doing this if the original data are heteroscedastic or multimodal. Furthermore, the size of the LDA-reduced subspace is limited to *c*-1 (where *c* is the number of classes).

Local Fisher's discriminant analysis (LFDA) combines the strategy of LDA with a locality-preserving projection to provide a linear manifold learning technique that preserves the within-class structure of the original space in the projected subspace; details of the LFDA algorithm applied in this study are provided in Sugiyama (2007). Briefly, LFDA seeks to find a transformation that preserves local neighborhood information, thereby ensuring that the underlying structure of the data distribution is preserved in the lower dimensional (size *r*) subspace. To accomplish this, the scatter matrices typical of LDA are scaled using an affinity matrix that measures the closeness of any two points relative to their *k_nn_*-nearest neighbor. The parameters *k_nn_* and *r* must be optimized in concert with the classifier for each subject. LFDA has been previously deployed as a preprocessing step for classification of walking intention (Kilicarslan et al., 2013) and classification of expressive movement (Cruz-Garza et al., 2014) from EEG. A similar locality preserving projection was also employed for detection of ankle movement intention from low frequency EEG (Xu et al., 2014).

#### Classification algorithm

Once a suitable algorithm for dimensionality reduction was determined, we next identified a classification scheme to decode movement intent from our EEG-based features. Gaussian mixture model (GMM) classifiers are common in the fields of biometrics and biomedical engineering because GMMs are capable of representing arbitrary statistical distributions as a weighted summation of multiple Gaussian distributions, termed components (Paalanen et al., 2006). Utilizing a GMM to compute the class-conditional probabilities in a maximum-likelihood classifier could improve performance over the traditional formulation, especially when the within-class feature set may be non-Gaussian, as could be the case for the temporally and spatially diverse EEG based features used in this study. The probability density function for a given training data set in the LFDA projected subspace, *X* = {*x_i_*}^n^_*i* = 1_ ∈ ℝ^*r*^, is given by:

(1) $$p(x)=\sum_{k=1}^{K}\alpha_{k}\phi_{k}$$

(2) $$\phi_{k}(x)=\frac{\exp[-0.5{\left(x-\mu_{k}\right)}^{\text{T}}\Sigma_{k}^{-1}(x-\mu_{k})]}{{\left(2\pi \right)}^{r/2}{\left|\Sigma_{k}\right|}^{1/2}}$$

where *K* is the number of components, α*_k_* is the mixing weight, μ*_k_* is the mean vector, and ∑_*k*_ is the covariance matrix of the *k*-th component. The parameters of each GMM component *K*, including α*_k_*, μ*_k_*, and ∑_*k*_, are estimated as those which maximize the log-likelihood of the training set given by:

(3) $$L_{k}=\sum_{i=1}^{n}\log p_{k}(x_{i})$$

where *p*(*x*) is given in (1). Maximization of (3) is carried out using an iterative expectation-maximization (EM) algorithm (Vlassis and Likas, 2002), with the initial estimate of the parameters α*_k_*, μ*_k_*, and ∑_*k*_ established via k-means clustering (Su and Dy, 2007), until the log-likelihood reaches a predetermined threshold. The number of components *K* is a critical parameter for successful implementation of a GMM classifier. During training, we limited the maximum value of *K* to be 10 and computed the maximum log likelihood from (3) for each model with values of *K* ∈ {1, 2 … 10}. We estimated the optimal value of *K* as the model that minimized the Bayes information criterion, which has been reported as an effective measure for optimizing the number of GMM components (Li et al., 2012). In this manner, GMMs representing each movement class were specified for use in a maximum-likelihood classifier.

The parameters for each class-conditional GMM were computed using an optimization data set for each participant (see Classifier optimization section). The parameter space which must be explored in order to fit these mixture models can be quite large, especially if the feature dimension is large. Given the limited time and training data available during EEG studies, this learning task may be impractical, but as indicated in the previous section, LFDA has been shown to effectively reduce data dimensionality while preserving the statistical information. Thus, we applied LFDA dimensionality reduction on our EEG feature set prior to training and testing a GMM model for use in a maximum-likelihood classifier of intended motion.

#### Classifier optimization

The EEG feature matrix from each trial was split into two mutually exclusive sets: one for LFDA-GMM classifier optimization and one for classifier evaluation (Figure 1). The optimization data set was selected randomly from the full data set, and it comprised 400 samples (2 s) of data from each class. The optimization data set was then split into two equally sized exclusive subsets, one for training and one for testing. The parameters for the LFDA-GMM classifier (the nearest neighbor (*k_nn_*) used in the affinity matrix, the dimensionality (*r*) of the projected subspace, and the number of mixture components (*K*) in the mixture model) were optimized for each subject and trial type—self initiated and triggered—using the optimization data set. Optimization involved three steps (Figure 1): (i) dimensionality reduction using LFDA for values of *k_nn_* and *r* from 1 to 249 and 1 to 250, (ii) identification of the optimal value of *K* for each class at each grid point in (i) using the training data from the optimization set, and (iii) computation of the accuracy of the LFDA-GMM classifier at each grid point in (i) using the testing data from the optimization set. The optimal parameters {*k_nn_*, *r*, *K*} for each subject were selected as those which produced the highest overall classification accuracy from the testing data.

#### Classifier performance via cross validation

The performance of the LFDA-GMM classifier with the optimal parameter set was analyzed for each subject and trial using repeated random sub-sampling cross validation (Figure 1). Repeated sub-sampling was chosen because the variable timing of the movements in each trial would result in an unequal number of samples from each class if *k-*fold cross validation scheme was used. The evaluation data set was randomly split into mutually exclusive training and testing data sets (Figure 1). Each of the three classes in the training set contained 600 data points representing 20% of the sit and stand classes. (Because the sit and stand classes were composed of ten 1.5 s long pre-movement epochs for each subject, their size was always equal). After training, LFDA-GMM classifier performance was analyzed using the testing data set, which contained all remaining data from the sit and stand classes, and an equal number of data points randomly selected from the quiet class. Thus, each class in the testing set contained 1900 data points. This test set structure was used to control for effects of class population size by assuring an equal number of testing samples in each class. During testing a classification decision was made for each data point, which represented a single time sample from the trial. The posterior probability of each data point was computed using the optimized GMM for each class and the data point was then assigned to the class that returned the largest value. This process yielded a classification decision for 1900 data points per trial. To avoid training bias, the random training and testing process was repeated 20 times and the average classification accuracies were reported for each subject under each condition (self-initiated and triggered movements). We performed *post-hoc* statistical comparisons between conditions using the non-parametric Kruskal-Wallis one-way analysis of variance.

To examine the effects of the ASR algorithm and the potential contribution of motion artifacts, we repeated the optimization and cross validation procedure using EEG data from pre-movement epochs pre-processed in the same manner as Figure 1 except that the ASR process was omitted. We also examined the classification accuracy using EEG epoched from movement onset to 1.5 s after movement onset both with and without the ASR algorithm. Finally, we divided the scalp into four major regions of interest (ROI) to assess the classification ability of each area individually. The ROIs included the frontal cortex (F3, F1, Fz, F2, F4, FC2, FC1, FC2, and FC4), the motor strip (C5, C3, C1, Cz, C2, C4, and C6), the parietal cortex (CP5, CP3, CP1, CPz, CP2, CP4, CP6, P3, P1, Pz, P2, and P4) and the central midline (FC1, FC2, C1, Cz, C2, CP1, CPz, and CP2). For each condition, we assessed within subject differences in accuracy across ROIs using the non-parametric Friedman test. The statistical sign test was used to assess if the difference in accuracy between self-initiated and triggered movements for each participant and ROI were significantly different from a distribution with a median of zero.

#### Demonstration of simulated real-time classification

We implemented a two-fold approach to demonstrate LFDA-GMM classifier performance in a simulated real-time environment using EEG data from the self-paced trial. The classifier was trained using ASR-cleaned EEG data from the first half of the trial with the optimal parameter set for each subject. Unlike during the cross-validation procedure, the time periods immediately following the movement execution were not trimmed from the data set but instead were included in the quiet class. Data from the second half of the trial, containing five transitions each of stand-to-sit and sit-to-stand, was used to test the controller in a simulated real-time manner resulting in a continuous time series of classification decisions.

### Observational EEG measures

In addition to classification of movement intent, we computed several observational measures to help assess differences in cortical activity across the experimental conditions. We computed the MRPs from each subject during both the self-initiated and triggered conditions. To compute MRPs, each EEG channel was band pass filtered between 0.1 and 50 Hz and epoched from 2.5 s before movement onset to 1 s after onset. Each channel and epoch was baseline corrected using the mean voltage from 2.5 to 2 s before onset. Each channel was then averaged over all 20 epochs for each condition.

To ascertain differences between periods of quiet (i.e., rest between movements), pre-movement, and post-movement epochs under each condition (self-initiated and triggered) we computed the power spectral density (PSD) for each EEG channel with a frequency resolution of 0.12 Hz using the Thompson Multitaper method in Matlab with a time bandwidth product of 4. The PSD was computed after artifact removal with ASR but before band-pass filtering and standardization. EEG was common average referenced for purposes of PSD computation. The spatial distribution of alpha band (8–13 Hz) ERD was computed for the pre-movement and post-movement epochs under both conditions as was the change in power in the delta band (0.1–4 Hz). The change in power for both frequency bands was computed relative to the quiet epochs for each condition (self-initiated and triggered). We assessed statistical differences across conditions using the non-parametric Kruskal-Wallis one-way analysis of variance with a Bonferroni correction for multiple comparisons.

## Results

### Observational measures

**Figure 2:**
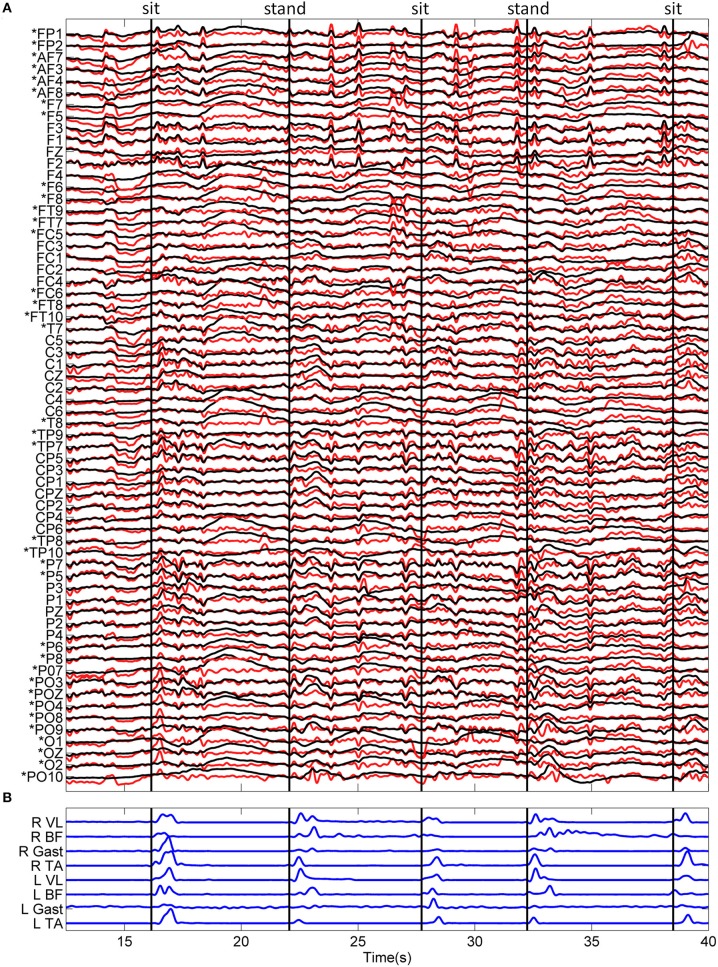
**Typical recordings of EEG and EMG data during the sitting and standing task. (A)** Standardized (z-score) EEG data is shown before (black) and after (red) ASR algorithm for artifact rejection. An asterisk (*) indicates peripheral channels which were removed prior to decoding. **(B)** The linear envelope of EMG data used to determine movement onset time, shown as vertical black lines. The type of movement is indicated at the top of the figure.

**Figure 3:**
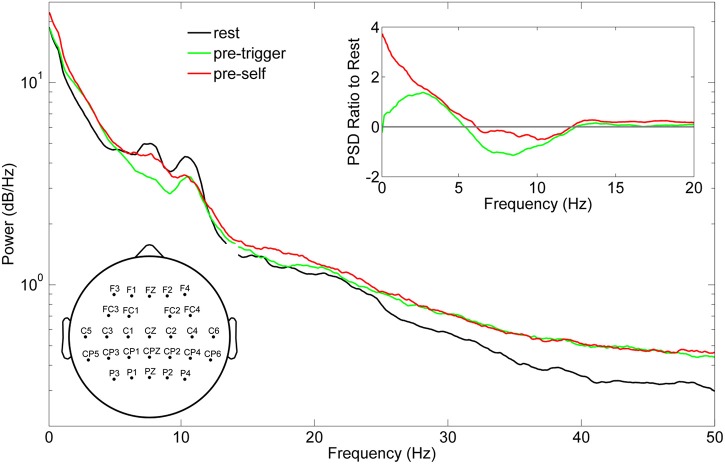
**Grand mean power spectral density (PSD) of EEG recordings across the 10 subjects**. The PSD was computed across all channels retained for neural decoding (left inset) during quiet standing (black line) and concatenated pre-movement epochs during triggered sitting and standing (pre-trigger, green line), and concatenated pre-movement epochs during self-initiated sitting and standing (pre-self, red line). The right inset shows the ratio of pre-trigger and pre-self PSD to rest.

Standardized EEG and the linear envelope of EMG recorded during a typical trial for one subject is shown in Figure 2. EEG with and without ASR is shown, demonstrating the removal of high amplitude artifacts, especially in the time periods following movement onset. Although all 64 channels of EEG are displayed, those channels marked with an asterisk (*) were removed prior to classification of movement intention. The EEG PSD computed during rest (quiet standing) and the pre-movement epochs during the self-initiated and triggered trials is shown in Figure 3. The grand mean PSD across all participants and electrodes used for classification (lower inset, Figure 3) is shown. Two identifiable peaks are present in the rest condition, during which the subject was standing quietly; one in the theta band at approximately 7 Hz and one in the alpha band at approximately 11 Hz. Power in these bands were significantly greater at rest than during the pre-movement epochs under both conditions (*p* < 0.01 for both). Notably, the delta band power during the pre-movement epochs was greater than rest while the power in the theta and alpha band was greater during rest (upper inset, Figure 3). In the pre-movement epochs, there was significantly less power in the theta band (4–8 Hz) during self-initiated transitions compared to triggered (*p* = 0.004), while power in the alpha band (8–13 Hz) was not statistically different between conditions (*p* = 0.107). Finally, power roll-off, indicated by the slope of the PSD, was diminished in theta and alpha bands compared to surrounding delta and beta bands for the self-initiated pre-movement; however, roll-off was only decreased in the alpha band for the triggered condition.

**Figure 4:**
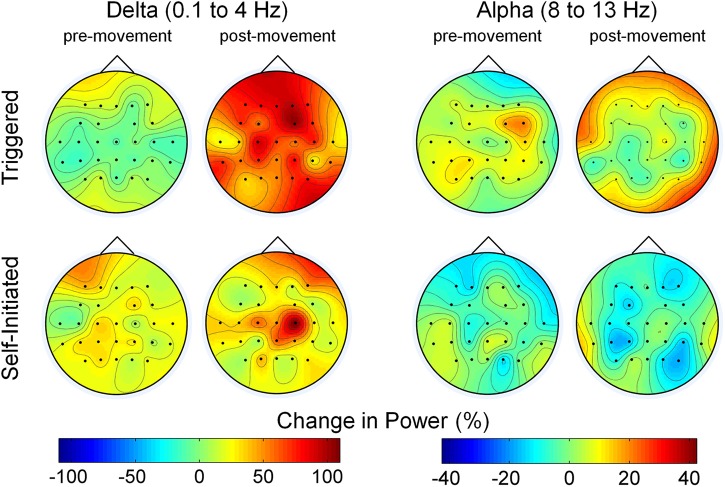
**Scalp maps of the change in power compared to rest during pre- and post-movement epochs**. The two sets of maps show the average change in delta and alpha band power across all electrodes and subjects during the pre-movement epoch (1.5 s before movement to movement onset) and post-movement epoch (movement onset to 1.5 s after onset) relative to the quiet state for both the triggered and self-initiated conditions.

The change in delta and alpha band power for the pre- and post-movement epochs, relative to the periods of quiet sitting and standing between movement executions, averaged over all participants is shown in Figure 4. In the delta band, we observed slightly increased power in the pre-movement epochs over all electrodes for both conditions, with slightly more delta power present in the self-initiated trials. In contrast, delta band power during the post-movement epochs was much larger, especially for the triggered trials, which showed nearly double the delta band power of the rest condition. The same level of increase was not observed over the full scalp in the self-initiated trials, although delta band power over the central midline electrodes increased by nearly 100%. Alpha band power was similar to quiet periods across most electrodes (note the difference in scale between alpha and delta power in Figure 4). Bilateral alpha band ERD was observed in both conditions; however for the triggered trials the ERD was less prominent and restricted to the central sensorimotor and parietal electrodes, while frontal and peripheral electrodes showed a slight increase in alpha power. Conversely, alpha ERD was stronger in the self-initiated condition, especially in the central-parietal areas of the scalp.

**Figure 5:**
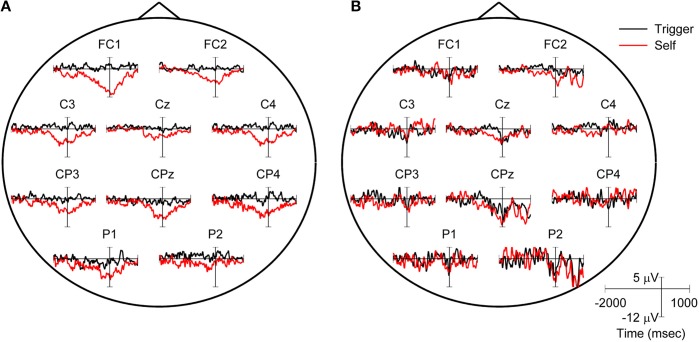
**Example of movement related potentials (MRPs) recorded in two different subjects. (A)** MRP from S5 indicating a difference between triggered (black line) and self-initiated (red line) movements. **(B)** MRP from S9 indicating similar, less prominent RPs for both the triggered and self-initiated trials. For each subject, MRPs were averaged across all 20 movements for each condition; movement onset is at 0 s.

We found the presence of MRPs to be variable across subjects and conditions. In 3 subjects, MRPs were prominent across the scalp during the self-initiated movement epochs but not during the triggered movements (Figure 5A). For the remaining subjects, less prominent MRPs were present at some electrodes for both conditions (Figure 5B). We examine the relationship between MRP and classification accuracy in more detail below.

### Classifier validation

**Figure 6:**
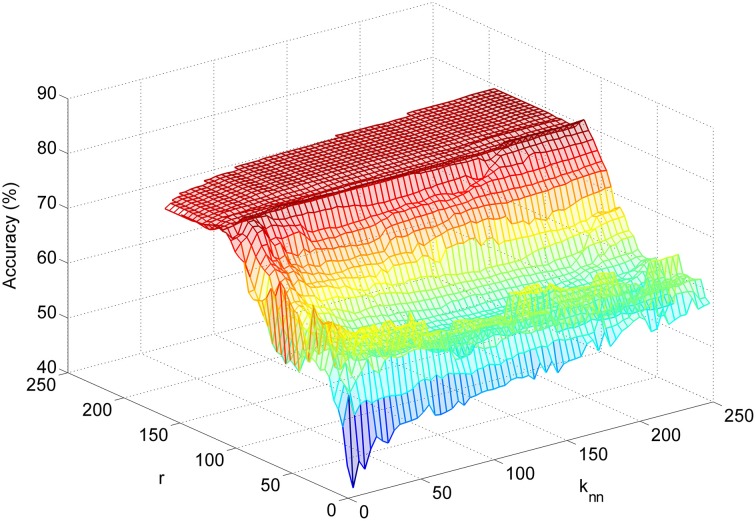
**Example of a subject-specific accuracy surface created during LFDA-GMM classifier optimization**. The accuracy plotted at each point {*r*, *k_nn_*} on the surface is the average accuracy with the optimal number of mixture components (*K*) for each class at that point.

The LFDA-GMM classification accuracy surface followed a similar pattern for most subjects (Figure 6), rising sharply as the size of the reduced subspace (*r*) increased. Accuracy typically peaked for *r* values between 50 and 125 before decreasing slightly, and then reaching a plateau as the value of *r* was further increased. Classification accuracy was generally insensitive to the *k_nn_* parameter with the exception of very low *r* values. The optimal parameter set for each subject and condition is provided in Table 1. Across subjects and conditions, the average dimension of the EEG-based feature space following LFDA was 88 (range 30–118), representing a significant reduction from the original size of 308. With few exceptions, the optimal accuracy was achieved using only one mixture component (*K* = 1) and thus, the LFDA-reduced EEG features were generally not strongly multimodal.

**Table 1:**
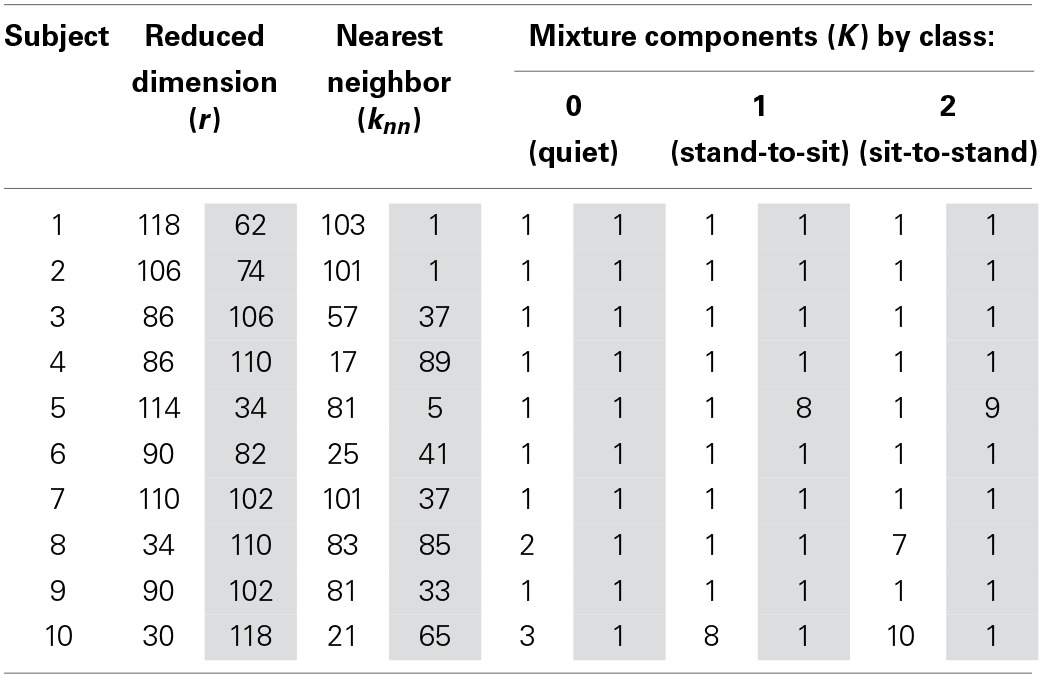
**Optimized LFDA-GMM parameters for each subject and condition**.

*The table indicates optimal parameter set for the triggered (white background) and self-initiated (shaded background) paradigms*.

**Figure 7:**
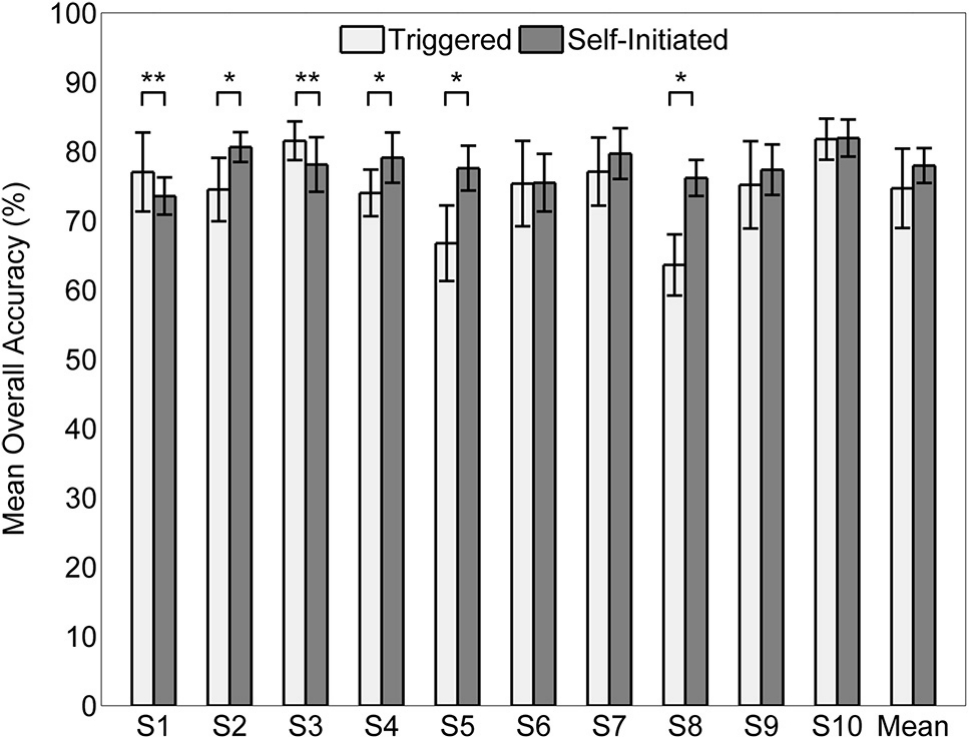
**Mean accuracy (*n* = 20) by subject for decoding triggered and self-initiated sitting and standing from pre-movement EEG**. Error bars indicate ±1 standard deviation. Statistically significant within subject differences across conditions are indicated as follows: ^*^p < 0.01, ^**^p < 0.05.

**Figure 8:**
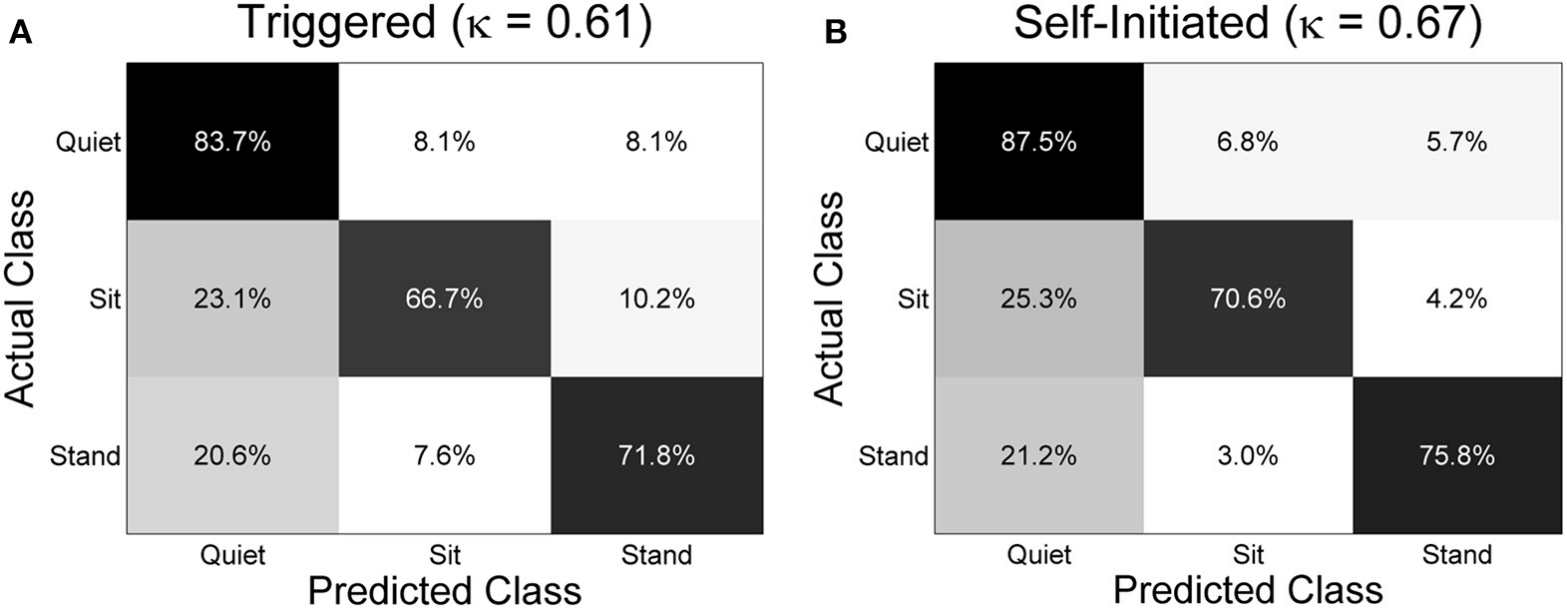
**Normalized confusion matrices across all subjects for the three class decoding problem for (A) triggered and (B) self-initiated conditions**. The confusion matrices were computed by totaling the predicted number of samples from each class across all 10 subjects and dividing by the total number of samples from each. For each repetition of the sub-sampling cross-validation procedure there were 1900 samples included in each class. The overall kappa coefficient for each condition is included in parentheses.

The mean overall classification accuracy obtained from the 20 times cross validation procedure for each subject and condition is shown in Figure 7 along with the overall mean across all subjects for each condition. The mean accuracy across subjects was 74.1 ± 5.7% for the triggered condition and 78.0 ± 2.6% for self-initiated. Testing sample size was equal across the three classes (1900 samples per class for each subject and condition). Interestingly, there was no significant difference in overall accuracy between self-initiated and triggered movements across the entire group of subjects. For subjects S2, S4, S5, and S7 decoding accuracy was significantly greater (*p* < 0.01) for the self-initiated sit-to-stand and stand-to-sit transitions compared to the triggered paradigm. Two subjects, S1 and S3, showed significantly better classification accuracy for the triggered movements compared to self-initiated, though with less strength (*p* < 0.05). The normalized confusion matrix for each condition was computed by summing the total number of predicted samples for each class across all 10 subjects and then dividing each predicted sum by the actual class sample size (Figure 8). We also computed the overall kappa coefficient (Cohen, 1968; Carletta, 1996) for each condition, resulting in κ = 0.61 for triggered and κ = 0.67 for self-initiated. For both triggered and self-initiated conditions, the quiet class was decoded with the highest accuracy and misclassifications for the quiet class were evenly distributed between the two types of movement (sit and stand). Notably, classification accuracy for all three classes was slightly, though not significantly, higher during the self-initiated trials. The majority of misclassifications for sit and stand movements were in the quiet class regardless of condition. Classifier confusion between movement types was slightly larger for the triggered paradigm, with 10.2% of sit movements misclassified as stand (as opposed to 4.2% for self-initiated) and 7.6% of stand movements misclassified as sit (compared to 3.0% for self-initiated).

**Figure 9:**
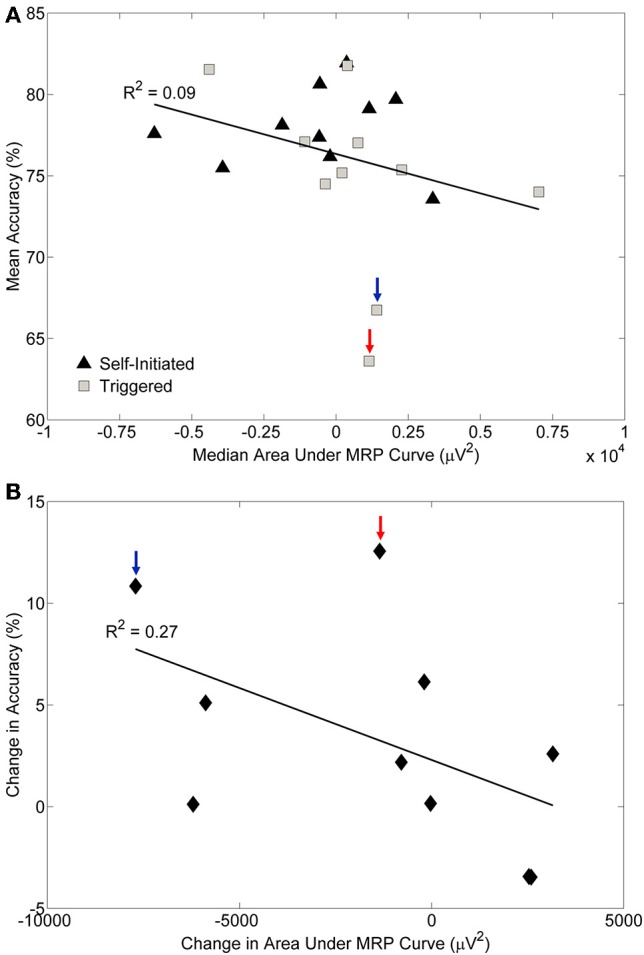
**Relationship between pre-movement decoding accuracy and the movement related potential (MRP)**. **(A)** The median area under the MRP curve plotted against the mean decoding accuracy for each subject and condition. A negative value of MRP area under the curve indicates the presence of larger MRPs. The coefficient of determination (R^2^) is indicated. **(B)** The change in decoding accuracy across conditions plotted against the change in area under the MRP curve for each subject. A large negative value for change in area indicates a stronger MRP presence during the self-initiated condition, while a large positive value indicates a stronger MRP presence during the triggered condition; values close to zero indicate similar MRPs for both conditions. The coefficient of determination (R^2^) is indicated. The two participants with the largest difference in accuracy across conditions are indicated by the arrows.

To assess the relationship between classifier accuracy and MRPs we computed the grand median area under the MRP curve for each condition and subject in a three step process. We first computed the area under the MRP of each channel for each movement epoch; a negative number for this area indicated a larger MRP presence. Next, we computed the median area under the curve for each electrode, and then we took the grand median area across all electrodes. We plotted this value against the mean classification accuracy for both the self-initiated and triggered conditions (Figure 9A). Surprisingly, we did not find a strong correlation between area under the MRP curve and classification accuracy (*R*^2^ = 0.09). Based on our prior observation that some subjects showed more prominent MRPs during the self-initiated movement compared to triggered, we computed the individual change in accuracy and the change in median area under the MRP curve across these conditions for each subject (Figure 9B). There was a slightly stronger, but still modest (*R*^2^ = 0.27) correlation between individual change in accuracy and area under the MRP curve. Interestingly, the subject with the most visually prominent difference in MRP between conditions (S5, Figure 5A; blue arrow in Figure 9B) showed the second largest increase in accuracy between the self-initiated and triggered conditions. However, the subject with the largest increase in accuracy across conditions (S8, red arrow in Figure 9B) showed only a moderate increase area under the MRP curve. The two subjects with significantly greater accuracy for the triggered condition also had larger areas under the MRP curve in that condition (Figure 9B).

### Classification by ROI

**Figure 10:**
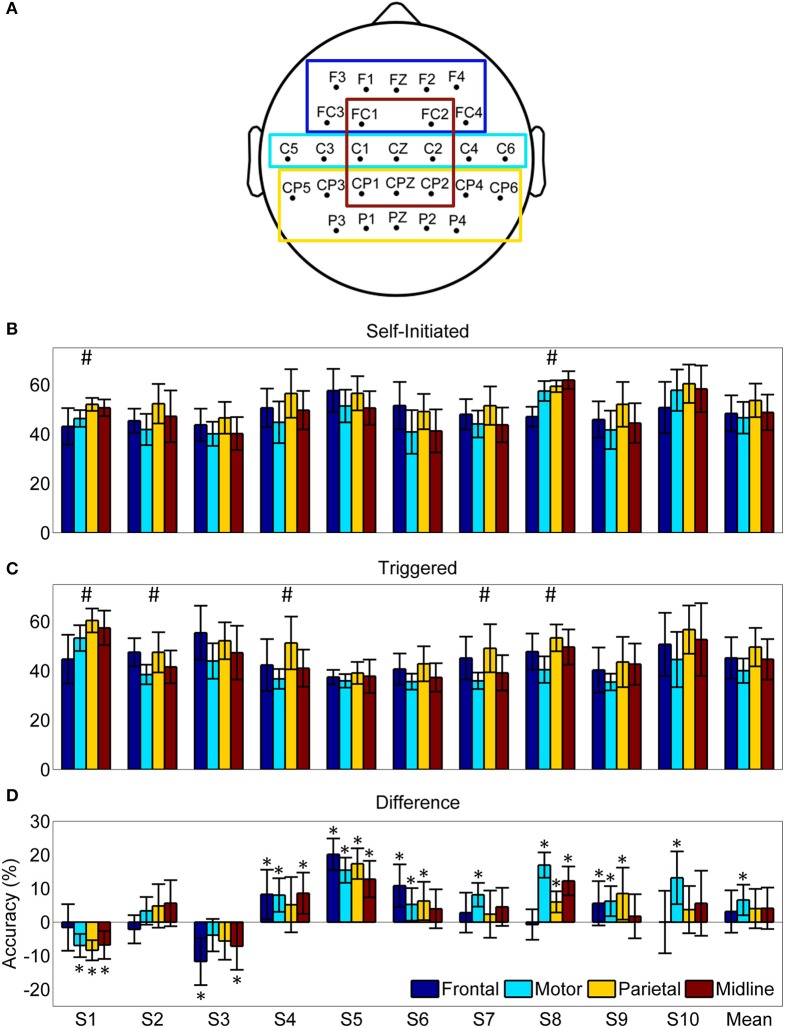
**Pre-movement decoding accuracy by region of interest (ROI)**. **(A)** Scalp map indicating the electrodes included in each ROI. **(B)** Average decoding accuracy ±1 standard deviation (*n* = 20) using the optimized LFDA-GMM algorithm for each ROI and subject during the self-initiated condition. **(C)** Average decoding accuracy ±1 standard deviation (*n* = 20) using the optimized LFDA-GMM algorithm for each ROI and subject during the triggered condition. Hash marks (#) indicates accuracy for at least one ROI is significantly different (*p* < 0. 05) for a given subject and condition based on Friedman's test. **(D)** The mean difference in pre-movement decoding accuracy between the self-initiated and triggered conditions for each subject ±1 standard deviation. Asterisks (*) indicate differences which were statistically significant (*p* < 0.05) from a distribution with a median of zero based on the sign test.

The mean and subject specific classification accuracy was lower for all four ROIs than with the full set of non-peripheral electrodes for both self-initiated and triggered movements (Figure 10), a result that was expected due to the lower number of electrodes used for classification. Of note, however, was that despite the differing number of electrodes within each ROI we observed few within subject significant differences in accuracy for each condition (Figures 10B,C). Similarly, when accuracy was averaged across the 10 subjects, there were no statistically significant differences between the ROIs for either condition. To assess the effect of self-initiated vs. triggered movements, we computed the within subject difference in accuracy for each ROI between these conditions (Figure 10D). A majority of participants (8/10) showed similar or significantly greater accuracy for all four ROIs in the self-initiated condition. The two subjects (S1 and S3) who showed significantly greater accuracy for the triggered movements with the full set of electrodes also showed greater accuracy in several, but not all, ROIs in this condition. Interestingly, when the difference was averaged across subjects, only the motor strip ROI showed significantly increased classification accuracy for the self-initiated condition. Indeed, decoding accuracy of movement intent during self-initiated sitting and standing using the motor ROI was significantly greater than during triggered movement in 7/10 subjects, similar in 2/10 subjects, and decreased in only 1/10 subjects.

### Effects of artifact removal

**Figure 11:**
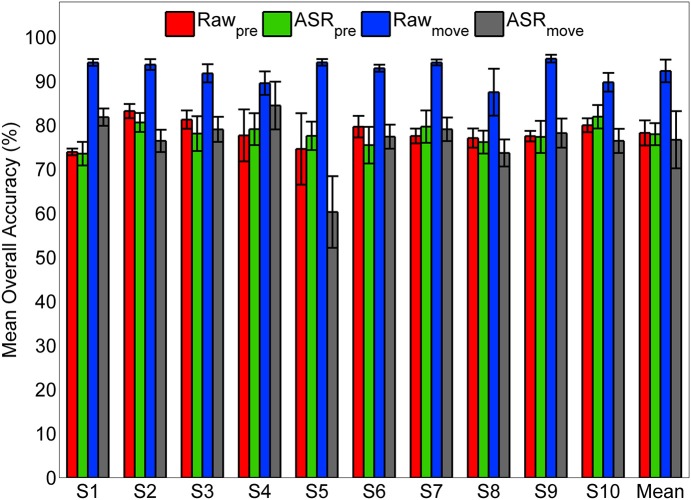
**Classification accuracy using pre- and post-movement epochs with and without ASR pre-processing**. The classifier was trained and tested for the self-initiated case using pre-movement epochs with the original pre-processing pipeline (ASR_pre_, green) and using pre-movement epochs omitting ASR from pre-processing (Raw_pre_, red). As a control, the classifier was also trained and tested using equally sized epochs (1.5 s) immediately following movement onset that were pre-processed with (ASR_move_, gray) and without (Raw_move_) ASR for artifact rejection.

To examine the effect of the ASR artifact rejection algorithm, and the potential effect of motion or other artifacts on classification accuracy, we repeated the classifier optimization and cross-validation procedure for the self-initiated condition using three control data sets and compared those with the original pre-processing (Figure 11). The original data set is termed ASR_pre_ in Figure 11. The first control data set was composed of the same pre-movement epochs consisting of 1.5 s of EEG data recorded immediately prior to movement onset, however, ASR was omitted from the pre-processing (Figure 1); this data set is termed Raw_pre_. We decoded movement intent using an equally sized epoch encompassing the 1.5 s time period immediately after movement onset. We processed these data with (ASR_move_) and without (Raw_move_) the ASR artifact rejection algorithm. We found that the ASR algorithm had no statistically significant affect on accuracy when using the pre-movement epochs to decode movement intent (Figure 11). This result was consistent for every subject and when accuracy was averaged across all subjects. When movement type was classified with EEG from epochs immediately after movement onset, a statistically significant increase in accuracy was observed in every subject when the data were not cleaned with ASR (Raw_move_). Application of the ASR algorithm (ASR_move_) resulted in a statistically significant drop in accuracy for decoding with the post-movement epochs in 9/10 subjects. When averaged across participants, no significant difference in accuracy was observed between ASR cleaned pre- and post-movement epochs, while accuracy was significantly higher for decoding with raw post-movement data.

### Simulated real-time classification

**Figure 12:**
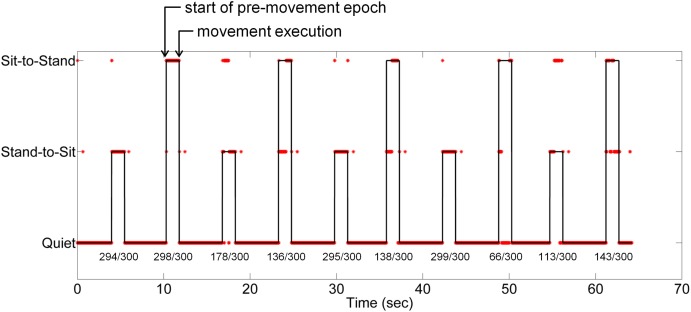
**Simulation of real-time decoding of movement intention from low frequency EEG for one subject**. The classifier was trained using ASR-cleaned EEG data from the first half of the self-initiated trial; the figure contains a time series of simulated real-time classification decisions from the second half of the trial. The line represents the true class of each time point; the asterisks show the LFDA-GMM classifier output. The percentage of correct decisions is provided under each stand-to-sit and sit-to-stand transition.

The results of simulated real-time decoding using cleaned EEG data are shown in Figure 12. Class-wise accuracy in this demonstration was different than observed from the cross-validation (Figure 8) an effect caused by the training sample bias inherent to the two-fold procedure used for the demonstration. The quiet class (0) contains a larger number of samples than either stand-to-sit (class 1) or sit-to-stand (class 2) resulting in very high accuracies during quiet periods. Confusion between classes 1 and 2 was present during most transitions; the low number of transitions used in this demonstration likely contributed to this confusion. Errors at the beginning and end of the movement periods skewed toward class 0 (quiet).

## Discussion

### Classification of self-initiated and triggered movement from pre-movement EEG

Our results demonstrate successful, high accuracy classification of movement intent in healthy individuals from delta-band EEG recorded before movement execution. We framed our experiment into a three-class problem where each time point was classified into one of three states: quiet, stand-to-sit transition, or sit-to-stand transition. It is important to note that we trimmed the time periods of actual movement execution—as determined from EMG activity—from our EEG recordings. Thus, our classifier was trained and tested using mutually exclusive EEG datasets recorded during either quiet standing or quiet sitting but when subjects presumably were preparing for the incoming action. We labeled each time point in the 1.5 s epoch before movement onset according to the type of movement that was executed in the future: stand-to-sit or sit-to-stand. All other time points were placed into a single quiet class. Classification ability was assessed in two different movement execution paradigms, one that was cued by an audio signal (triggered) and one that was self-paced (self-initiated). Interestingly, we observed no statistically significant difference in classification accuracy between these two conditions, though average accuracy across the 10 subjects was slightly higher for the self-initiated condition (78.0 ± 2.6%) compared to triggered (74.7 ± 5.7%) and both of these were significantly better than chance accuracy of 33.3%.

Prominent MRPs were not visible in all subjects (Figure 5) and we found almost no correlation between median area under the MRP curve and classification accuracy (Figure 9A). For within subject comparisons between conditions, we observed significantly better accuracy in four of ten subjects during the self-initiated compared to triggered paradigm, while two subjects had higher accuracy for triggered standing and sitting. When examining subject specific changes in accuracy across the two different paradigms, we found a slightly stronger correlation between increased accuracy and area under the MRP curve. And the two individuals that showed a decrease in accuracy in the self-initiated vs. triggered trials also showed an increased area under MRP curve, indicating less prominent MRPs. These results appear to contradict previous examples which indicated that MRPs may be more prominent in self-paced vs. cued movement paradigms (Jahanshahi et al., 1995; Jankelowitz and Colebatch, 2002; Cui and MacKinnon, 2009). There are several possible explanations. First, our experimental paradigm included a relatively low number of epochs (*n* = 20) for each condition, compared to traditional studies of MRPs which typically utilize close to 100 (Shibasaki and Hallett, 2006). This low number of epochs may be the reason for the large variability in the presence of MRPs (Figure 5). Additionally, in the self-paced experiment, participants were instructed to pause 3–10 s between each movement though they were also instructed not to count the seconds between each movement. As a result, participants rarely waited 10 s between self-paced movements; most periods of quiet lasted 5 s or less. Previous studies have observed trial-to-trial variation in timing and power of MRPs relating to self-paced left and right hand movements, making classification of those movements using low frequency features more difficult (Bai et al., 2007). Another study found that while they were present for most—but not all—subjects and movements, low frequency features were less critical than ERD/ERS in classifying four different types of movement from EEG (Morash et al., 2008). The latter study utilized the contingent negative variation (CNV), which is a low frequency, event related-potential entailing a widespread negative shift in EEG observed in paradigms involving conditional and imperative stimuli (Walter et al., 1964). While our paradigm did not involve dual stimuli, it is possible that some participants experienced a similar effect due to the alternating nature of the movements. That is, completing the previous maneuver (sitting or standing) may have created a conditional response in which the subject then began to prepare for the next movement, which would be the opposite of the prior one. This conditional response may be another reason that we did not observe prominent MRPs in some subjects. Indeed, trial-to-trial variation in CNV amplitude has been described previously and this variation may be representative of anticipated events and/or fluctuations in attention to the task (Scheibe et al., 2010). The observed variation in MRPs may also be responsible for the skewed misclassification of sit and stand movement intentions as quiet (Figure 8). Note that while the full time series of EEG data contained more samples in the “quiet” class than in the “sit” and “stand” class, an equal amount of data from each class was used for cross-validation, and thus, this pattern of misclassification was not a result of training bias.

Variable timing of movement execution and conditional response may have affected the prominence of MRPs, but it did not hinder classification accuracy. One reason for this may be the time-embedding of our classification features which encompassed information from up to 50 ms before the current time point, helping to alleviate previously reported MRP-based feature variability (Bai et al., 2007). Low frequency EEG has been shown to contain information regarding intention (Lew et al., 2012), direction (Liao et al., 2007; Vuckovic and Sepulveda, 2008; Waldert et al., 2008; Robinson et al., 2013), velocity (Bradberry et al., 2010), and type (Agashe and Contreras-Vidal, 2013) of hand movement. In the lower extremity, the ability to detect voluntary ankle dorsiflexion movement from MRPs with accuracies up to 80% has been reported (Niazi et al., 2011; Xu et al., 2014). During walking, intra-stride changes in electrocortical activity coupled to gait phase have been observed at frequencies as low as 3 Hz (Gwin et al., 2011) and inter-limb and intra-limb kinematics (Presacco et al., 2011, 2012) as well as the intention to start and stop walking (Kilicarslan et al., 2013) have been decoded using delta band EEG. In another recent study, features extracted from the delta band were the most heavily weighted for single trial classification of walking movement intention from EEG recorded prior to movement (Velu and de Sa, 2013). Our results, which classified lower extremity movement type using pre-movement EEG, corroborate these findings and provide further evidence that low frequency EEG contains discriminative information pertaining to lower extremity movement intent.

### Classification by region of interest

The results from our ROI analysis (Figure 10) support the hypothesis that stand-to-sit and sit-to-stand transitions are preceded by event-related activity across a distributed, sparse cortical network. As expected due to the reduced number of electrodes, no ROI reached the classification accuracy attained when all electrodes were included in the classifier. When averaged across subjects, there were no statistically significant differences in classification accuracy between the ROIs for either condition, despite the difference in number of electrodes. The ROI analysis also revealed a statistically significant increase in accuracy for within subject differences across conditions (self-initiated vs. triggered) when using only the electrodes over the motor area. A similar difference was not found for any other ROI or for the entire scalp. This result suggests that the primary motor cortex (M1) region contains more discriminative information for identification of standing and sitting intention when the movements are self-initiated compared to cued. This finding is supported by previous work indicating MRPs from this region differ when the motor task emphasized sequence initiation compared to rhythm (Bortoletto et al., 2011). EEG recorded from these electrodes has also been demonstrated to most accurately track movement initiation using other frequency bands such as mu/alpha ERD and beta ERS (Wolpaw et al., 2002).

### Artifact subspace reconstruction

This study, along with previously mentioned work, establishes compelling evidence for neural correlates of movement within EEG signals recorded immediately prior to movement execution; however, it is important to address the possible role of artifacts, both physiological such as muscle and eye and non-physiological, such as movement. Our signal processing approach for classifier training and evaluation (Figure 1) was designed to minimize the effect of artifacts in several ways. First, we eliminated frontal, temporal, and occipital electrodes which can be contaminated by EMG and/or EOG artifacts. Second, we trimmed all EEG that was recorded during periods of movement as indicated by lower extremity EMG from our data set, leaving only EEG recorded during periods of quiet sitting and standing for classification. Third, we applied a PCA-based artifact rejection algorithm (ASR) that was designed to eliminate high amplitude and high variance artifacts, such as those from movement or muscle, from EEG (Mullen et al., 2013). Our pre-processing analysis demonstrated similar power spectral density between rest (quiet standing) and pre-movement periods under both conditions (Figure 3), suggesting that our pre-processing steps were effective in removing artifacts from EEG. We also observed alpha ERDs in the period immediately following movement onset (Figure 4), especially during self-initiated trials, an observation that would have been unlikely if muscle activity had remained in the cleaned-EEG signals since EMG tends to have power in this frequency band.

To further elucidate the possible role of artifacts and these steps to mitigate them, we compared the LFDA-GMM classifier performance when it was trained and tested with three different control data sets with our original processing pipeline (Figure 11). This analysis showed no statistically significant difference in accuracy, regardless of whether the pre-movement EEG was cleaned with ASR or not, suggesting that artifacts were not present and therefore did not affect classification using the pre-movement epochs. We did observe a significant increase in accuracy when the pre-movement epochs were replaced with equally sized epochs immediately following movement onset that had not been cleaned using ASR. After ASR cleaning, classification accuracy was commensurate with pre-movement epochs, although with a slightly larger standard deviation across subjects. The increased accuracy using post-movement epochs without ASR suggests that artifacts may have been present during this time and these artifacts may have enhanced decoding accuracy. The decreased accuracy following ASR suggests that this algorithm is effective at removing high amplitude artifacts from EEG data. This conclusion is further supported by the simulated real-time demonstration using ASR-cleaned data. The time periods after movement onset were included in the quiet class during training and were decoded with high accuracy during testing (Figure 12). But, caution should be exercised regarding the conclusion that ASR completely eliminates low frequency, high amplitude artifacts. We note that while we did observe alpha ERD in ASR-cleaned post-movement epochs, we also observed enhanced power in the delta band across the scalp, particularly in the triggered condition (Figure 4). One possible explanation for the post-movement increase in delta band power in the triggered trials could be residual head movement and/or muscle artifacts as the participant reacted to the audio cue to stand or sit. Further spectral, topographical, and temporal analysis should be undertaken to parse movement related artifacts from true electrocortical sources recording during the actual sitting and standing movements. In particular, the parameters of the ASR algorithm can be optimized to more aggressively remove artifacts at the expense of potentially removing true EEG. We emphasize that our primary analysis involved only EEG from pre-movement and quiet periods, thereby limiting the contribution of these potential artifactual components as indicated by the above analysis.

### EEG use in rehabilitation and restoration of movement

To our knowledge, this is the first study that classifies this type of gross, full lower extremity movement intention—sit-down, stand-up, or quiet—from non-invasive EEG signals. Previously, surface EMG from leg muscles has been used with an LDA classifier to identify standing and sitting transition in amputees with accuracies greater than 99% (Zhang et al., 2012). Achievement of these high accuracies required the use of a post-processing majority voting step, which resulted in a decision delay of up to 400 ms. Another approach has deployed center of pressure to detect sitting and standing transition in individuals with paraplegia (Quintero et al., 2011). Classification of sitting and standing using EEG offers advantages over these approaches. On average, we were able to achieve 78% accuracy using features extracted from the pre-movement epochs with no post-processing required, thereby minimizing delay between movement intention and classification. It should be noted that our classification accuracy was assessed using single time points that were randomly selected from each trial. This conservative approach was necessary to prevent model over-fitting during training and to assure an equal number of data points in each class during testing due to the relatively low number of movements executed (20 per condition) for each subject. An example of the LFDA-GMM algorithm in a simulated real-time environment is shown in Figure 12. We note that classifier training was not optimal for this demonstration; only 5 stand-to-sit and sit-to-stand transitions were employed. Further, clinical deployment of the classifier as a component of a BMI could be significantly improved by addition of an aggregate post-processing step—such as requiring a number of consecutive time points to be predicted as the same movement type or a sliding window moving average with a threshold—to trigger a change in state. The parameters of this post-processing step need to be tuned for each subject and application to maximize accuracy and minimize false positives. Future studies will investigate this possibility and the tradeoff between gains in accuracy and increased classification latency from post-processing.

One drawback of utilizing GMM based classifiers is the size of the parameter space which must be learned, which is given by *K* * (1 + *d* * (*d* − 1)/2) + *K* * *d*, where *K* is the number of Gaussian components in the mixture, and *d* is the dimensionality of the data to be fit (Li et al., 2012). To fit a GMM to our time-embedded EEG-based feature data set, which includes data from 28 channels of EEG at 11 time points and a maximum of *K* = 10 components for a given class, requires learning a parameter space of dimension 4.76 × 10^5^. Our results demonstrate that LFDA is a powerful dimensionality reduction technique; the median dimension of the reduced subspace was 96 (Table 1), representing a median reduction of 69% across subjects. LFDA reduced the size of the GMM parameter by an order of magnitude, resulting in a large decrease of computation time to fit the models of the classifier. Classifier optimization and training was performed using custom software developed in Matlab®, including the parallel processing toolbox, run on a dual core PC (2.40 GHz, 24 GB RAM). On average, optimization across the full LFDA-GMM parameter space was complete in less than 15 min per subject, and training of the optimized LFDA-GMM classifier in less than 5. If deployed for control of an assistive device, LFDA-GMM classifier optimization and training may be required before each session of use; these results suggest this is feasible. Examination of the optimization surface (Figure 6) shows that gains in accuracy level-off at moderate values of *r* while accuracy is relatively insensitive to *k_nn_*. The same trend is observed in all subjects, with some showing decreases in accuracy for increasing *r*-values, while in others there is no difference in accuracy as the parameter values are increased. Thus, these parameters could be limited to smaller values, thereby reducing the parameter space to be searched during LFDA-GMM optimization. However, the optimal parameter set is expected to vary with the task and also with the ability of the subject to learn how to operate the BMI over time, and so caution should be exercised when determining the upper limits. Also, full covariance matrices (∑_*k*_) were deployed for each component of the GMMs; however, if the subspace of the data following LFDA dimensionality reduction was large, employing diagonal covariance matrices could be used as a way to speed classifier training.

The LFDA-GMM classifier presented here could be incorporated into a closed loop BMI system with an exoskeleton to restore function to individuals with paralysis. Such a system would be comprised of a shared control paradigm, whereby the gross motor instruction (in this case, the intention to sit-down or stand-up) is extracted from the user's EEG and the commands to execute the movement are performed autonomously by the exoskeleton. In this setup, the exoskeleton would be triggered at the first time point in which the BMI detected a change in class; a process that would likely include a post-processing step requiring a sequence of consistent classifier decisions to trigger a change in state. The decoding algorithm would then be blanked so that no state changes could be triggered during the execution of a movement. Our observed accuracy of 78% in self-paced movements would need to be improved for clinical viability. However, the data used in this study were purely observational, while operation of a BMI is a learned skill that incorporates feedback to the user regarding performance; thus accuracy of the BMI may increase as the user gains additional experience with the device. In the future, EEG and EMG could be combined to create a comprehensive neural-machine interface for control of advanced prosthetics. The combined EEG-EMG interface could provide intuitive control of artificial limbs while minimizing delay between detection of voluntary movement intention and its execution. Our classification approach could also be used in an intervention to treat phantom limb pain, whereby a descending motor command is determined from EEG and a motorized prosthesis executes the movement providing afferent feedback which could obviate maladaptive cortical reorganization following amputation. EEG-based classification of movement intent could also be incorporated into a neurorehabilitation protocol to recover more normal motor function in individuals with neurologic impairments. For example, the EEG based classifier would activate a device to assist movement, thereby creating more normal afferent feedback, which could enhance brain plasticity and speed motor recovery (Daly and Wolpaw, 2008). Such a strategy requires extraction of motion intent from the motor impaired population; in this study only healthy able-bodied individuals were tested. Future studies will examine the ability to apply LFDA-GMM classification to individuals with central nervous systems deficits with an aim toward neurorehabilitation strategies.

### Conflict of interest statement

The authors declare that the research was conducted in the absence of any commercial or financial relationships that could be construed as a potential conflict of interest.

## References

[B1] Agashe H. A. Contreras-Vidal J. L. (2013). Decoding the evolving grasping gesture from electroencephalographic (EEG) activity, in Proceedings 35th International Conference IEEE Engineering Medicine Biology Society (Osaka), 5590–5593. 10.1109/EMBC.2013.6610817 PMC3801391 24111004

[B2] Bai O. Lin P. Vorbach S. Li J. Furlani S. Hallett M. (2007). Exploration of computational methods for classification of movement intention during human voluntary movement from single trial EEG. Clin. Neurophysiol. 118, 2637–2665. 10.1016/j.clinph.2007.08.025 17967559 PMC4154235

[B3] Bai O. Rathi V. Lin P. Huang D. Battapady H. Fei D. Y.. (2011). Prediction of human voluntary movement before it occurs. Clin. Neurophysiol. 122, 364–372. 10.1016/j.clinph.2010.07.010 20675187 PMC5558611

[B4] Ball T. Schulze-Bonhage A. Aertsen A. Mehring C. (2009). Differential representation of arm movement direction in relation to cortical anatomy and function. J. Neural Eng. 6:016006. 10.1088/1741-2560/6/1/016006 19155551

[B5] Bashashati A. Fatourechi M. Ward R. K. Birch G. E. (2007). A survey of signal processing algorithms in brain-computer interfaces based on electrical brain signals. J. Neural. Eng. 4, R32–R57. 10.1088/1741-2560/4/2/R03 17409474

[B6] Beuchat N. J. Chavarriaga R. Degallier S. Millan J. R. (2013). Offline decoding of upper limb muscle synergies from EEG slow cortical potentials, in 35th Annual International Conference IEEE Engineering Medicine Biology Society (Osaka), 3594–3597. 10.1109/EMBC.2013.6610320 24110507

[B7] Birbaumer N. Ghanayim N. Hinterberger T. Iversen I. Kotchoubey B. Kubler A.. (1999). A spelling device for the paralysed. Nature 398, 297–298. 10.1038/18581 10192330

[B8] Boord P. Craig A. Tran Y. Nguyen H. (2010). Discrimination of left and right leg motor imagery for brain-computer interfaces. Med. Biol. Eng. Comput. 48, 343–350. 10.1007/s11517-010-0579-0 20143173

[B9] Bortoletto M. Cook A. Cunnington R. (2011). Motor timing and the preparation for sequential actions. Brain Cogn. 75, 196–204. 10.1016/j.bandc.2010.11.016 21190763

[B10] Boschert J. Deecke L. (1986). Cerebral potentials preceding voluntary toe, knee and hip movements and their vectors in human precentral gyrus. Brain Res. 376, 175–179. 10.1016/0006-8993(86)90913-3 3719365

[B11] Bradberry T. J. Gentili R. J. Contreras-Vidal J. L. (2010). Reconstructing three-dimensional hand movements from noninvasive electroencephalographic signals. J. Neurosci. 30, 3432–3437. 10.1523/JNEUROSCI.6107-09.2010 20203202 PMC6634107

[B12] Brunia C. H. M. Van Den Bosch W. E. J. (1984). Movement-related slow potentials I: a contrast between finger and foot movements in right-handed subjects. Electroenceph. Clin. Neurophysiol. 57, 515–527. 10.1016/0013-4694(84)90087-7 6202482

[B13] Bulea T. C. Kilicarslan A. Ozdemir R. Paloski W. H. Contreras-Vidal J. L. (2013). Simultaneous scale electroencephalography (EEG), electromyography (EMG), and whole-body segmental inertial recording for multi-modal neural decoding. J. Vis. Exp. 77, e50602. 10.3791/50602 23912203 PMC3846438

[B14] Carletta J. (1996). Assessing agreement on classification tasks: the kappa statistic. Comput. Linguist. 22, 249–254.

[B15] Castermans T. Duvinage M. Cheron G. Dutoit T. (2014). About the cortical origin of the low-delta and high-gamma rhythms observed in EEG signals during treadmill walking. Neurosci. Lett. 561, 166–170. 10.1016/j.neulet.2013.12.059 24412128

[B16] Cohen J. (1968). Weighted kappa: nominal scale agreement provision for scaled disagreement or partial credit. Psychol. Bull. 70, 213–220. 10.1037/h0026256 19673146

[B17] Cruz-Garza J. G. Hernandez Z. R. Nepaul S. Bradley K. K. Contreras-Vidal J. L. (2014). Neural decoding of expressive human movement from scalp electroencephalography (EEG). Front. Hum. Neurosci. 8:188. 10.3389/fnhum.2014.00188 24782734 PMC3986521

[B18] Cui R. MacKinnon C. D. (2009). The effect of temporal accuracy constraints on movement-related potentials. Exp. Brain Res. 194, 477–488. 10.1007/s00221-009-1725-5 19221724

[B19] Daly J. J. Wolpaw J. R. (2008). Brain-computer interfaces in neurological rehabilitation. Lancet Neurol. 7, 1032–1043. 10.1016/S1474-4422(08)70223-0 18835541

[B20] Delorme A. Makeig S. (2004). EEGLAB: an open source toolbox for analysis of single-trial EEG dynamics. J. Neurosci. Methods 134, 9–21. 10.1016/j.jneumeth.2003.10.009 15102499

[B21] Delorme A. Sejnowski T. Makeig S. (2007). Enhanced detection of artifacts in EEG data using higher-order statistics and independent component analysis. Neuroimage 34, 1443–1449. 10.1016/j.neuroimage.2006.11.004 17188898 PMC2895624

[B22] do Nascimento O. F. Farina D. (2008). Movement-related cortical potentials allow discrimination of rate of torque development in imaginary isometric plantar flexion. IEEE Trans. Biomed. Eng. 55, 2675–2678. 10.1109/TBME.2008.2001139 18990639

[B23] do Nascimento O. F. Nielsen K. D. Voigt M. (2005). Influence of directional orientations during gait initiation and stepping on movement-related cortical potentials. Behav. Brain Res. 161, 141–154. 10.1016/j.bbr.2005.02.031 15904721

[B24] do Nascimento O. F. Nielsen K. D. Voigt M. (2006). Movement-related parameters modulate cortical activity during imaginary isometric plantar-flexions. Exp. Brain Res. 171, 78–90. 10.1007/s00221-005-0247-z 16320044

[B25] Gallivan J. P. McLean D. A. Valyear K. F. Pettypiece C. E. Culham J. C. (2011). Decoding action intentions from preparatory brain activity in human parieto-frontal networks. J. Neurosci. 31, 9566–9610. 10.1523/JNEUROSCI.0080-11.2011 21715625 PMC6623162

[B26] Garrett D. Peterson D. A. Anderson C. W. Thaut M. H. (2003). Comparison of linear, nonlinear, and feature selection methods for EEG signal classification. IEEE Trans. Neural Syst. Rehab. 11, 141–144. 10.1109/TNSRE.2003.814441 12899257

[B27] Gwin J. T. Gramann K. Makeig S. Ferris D. P. (2010). Removal of movement artifact from high-density EEG recorded during walking and running. J. Neurophysiol. 103, 3526–3534. 10.1152/jn.00105.2010 20410364 PMC3774587

[B28] Gwin J. T. Gramann K. Makeig S. Ferris D. P. (2011). Electrocortical activity is coupled to gait cycle phase during treadmill walking. Neuroimage 54, 1289–1296. 10.1016/j.neuroimage.2010.08.066 20832484

[B29] Hallett M. (1993). Movement-related cortical potentials. Electromyogr. Clin. Neurophysiol. 34, 5–13. 8168458

[B30] Hodges P. W. Bui B. H. (1996). A comparison of computer-based methods for the determination of onset of muscle contraction using electromyography. Electroenceph. Clin. Neurophysiol. 101, 511–519. 10.1016/S0013-4694(96)95190-5 9020824

[B31] Hogan N. Krebs H. I. (2011). Physically interactive robotic technology for neuromotor rehabilitation. Prog. Brain Res. 192, 59–68. 10.1016/B978-0-444-53355-5.00004-X 21763518

[B32] Hung C. I. Lee P. L. Wu Y. T. Chen L. F. Yeh T. C. Hsieh J. C. (2005). Recognition of motor imagery electroencephalography using independent component analysis and machine classifiers. Ann. Biomed. Eng. 33, 1053–1070. 10.1007/s10439-005-5772-1 16133914

[B33] Ikeda A. Lüders H. O. Burgess R. C. Shibasaki H. (1992). Movement-related potentials recorded from supplementary motor area and primary motor area. Brain 115, 1017–1043. 10.1093/brain/115.4.1017 1393500

[B34] Jahanshahi M. Jenkins H. Brown R. G. Marsden D. Passingham R. E. Brooks D. J. (1995). Self initiated versus externally triggered movements I: an investigation using measurement of regional cerebral blood flow with PET and movement-related potentials in normal and Parkinson's disease subjects. Brain 118, 913–933. 10.1093/brain/118.4.913 7655888

[B35] Jankelowitz S. K. Colebatch J. G. (2002). Movement-related potentials associated with self-paced, cued and imagined arm movements. Exp. Brain Res. 147, 98–107. 10.1007/s00221-002-1220-8 12373374

[B36] Jiang N. Gizzi L. Mrachacz-Kersting N. Dremstrup K. Farina D. (2014). A brain-computer interface for single trial detection of gait initiation from movement related cortical potentials. Clin. Neurophysiol. [Epub ahead of print]. 10.1016/j.clinph.2014.05.003 24910150

[B37] Kilicarslan A. Prasad S. Grossman R. G. Contreras-Vidal J. L. (2013). High accuracy decoding of user intentions using EEG to control a lower-body exoskeleton, in 35th Annual International Conference IEEE Engineering Medicine Biology Society (Osaka), 5606–5609. 10.1109/EMBC.2013.6610821 PMC3801445 24111008

[B38] Lew E. Chavarriaga R. Silvoni S. Millan J. R. (2012). Detection of self-paced reaching movement intention from EEG signals. Front. Neuroeng. 5:13. 10.3389/fneng.2012.00013 23055968 PMC3458432

[B39] Li W. Prasad S. Fowler J. Bruce L. M. (2012). Locality-preserving dimensionality reduction and classification for hyperspectral image analysis. IEEE Trans. Geosci. Rem. Sens. 50, 1185–1198 10.1109/TGRS.2011.2165957

[B40] Li X. Zhou P. Aruin A. S. (2007). Teager-Kaiser energy operation of surface EMG improves muscle activity onset detection. Ann. Biomed. Eng. 35, 1532–1538. 10.1007/s10439-007-9320-z 17473984

[B41] Liao X. Yao D. Wu D. Li C. (2007). Combining spatial filters for the classification of single-trial EEG in a finger movement task. IEEE Trans. Biomed. Eng. 54, 821–831. 10.1109/TBME.2006.889206 17518278

[B42] Liu J. Perdoni C. He B. (2011). Hand movement decoding by phase-locking low frequency EEG signals, in Proceedings 33rd International Conference IEEE Engineering Medicine Biology Society (Boston, MA), 6335–6338. 10.1109/IEMBS.2011.6091564 22255787

[B43] Lotte F. Congedo M. Lecuyer A. Lamarche F. Arnaldi B. (2007). A review of classification algorithms for EEG-based brain-computer interfaces. J. Neural Eng. 4, R1–R13. 10.1088/1741-2560/4/2/R01 17409472

[B44] Millán J. R. Renkens F. Mourino J. Gerstner W. (2004). Noninvasive brain-actuated control of a mobile robot by human EEG. IEEE Trans. Biomed. Eng. 51, 1026–1033. 10.1109/TBME.2004.827086 15188874

[B45] Morash V. Bai O. Furlani S. Lin P. Hallett M. (2008). Classifying EEG signals preceding right hand, left hand, tongue, and right foot movements and mtoor imageries. Clin. Neurophysiol. 119, 2570–2578. 10.1016/j.clinph.2008.08.013 18845473 PMC2602863

[B46] Mrachacz-Kersting N. Kristensen S. R. Niazi I. K. Farina D. (2012). Precise temporal association between cortical potentials evoked by motor imagination and afference induces cortical plasticity. J. Physiol. 590, 1669–1682. 10.1113/jphysiol.2011.222851 22250210 PMC3413497

[B47] Mullen T. Kothe C. Chi Y. M. Ojeda A. Kerth T. Makeig S.. (2013). Real-time modeling and 3D visualization of source dynamics and connectivity using wearable EEG, in 35th International Conference IEEE Engineering Medicine Biology Society (Osaka), 2184–2187. 10.1109/EMBC.2013.6609968 PMC4119601 24110155

[B48] Muller K. R. Anderson C. W. Birch G. E. (2003). Linear and nonlinear methods for brain-computer interfaces. IEEE Trans. Neural Syst. Rehab. 11, 165–169. 10.1109/TNSRE.2003.814484 12899264

[B49] Niazi I. K. Jiang N. Jochumsen M. Nielsen J. F. Dremstrup K. Farina D. (2013). Detection of movement-related cortical potentials based on subject-independent training. Med. Biol. Eng. Comput. 51, 507–512. 10.1007/s11517-012-1018-1 23283643 PMC3627050

[B50] Niazi I. K. Jiang N. Tiberghien O. Nielsen J. F. Dremstrup K. Farina D. (2011). Detection of movement intention from single-trial movement related cortical potentials. J. Neural Eng. 8:066009. 10.1088/1741-2560/8/6/066009 22027549

[B51] Niazi I. K. Mrachacz-Kersting N. Jiang N. Dremstrup K. Farina D. (2012). Peripheral electrical stimulation triggered by self-paced detection of motor intention enhances motor evoked potentials. IEEE Trans. Neural Syst. Rehab. Eng. 20, 595–604. 10.1109/TNSRE.2012.2194309 22547461

[B52] Paalanen P. Kamarainen J. K. Ilonen J. Kalviainen H. (2006). Feature representation and discrimination based on Gaussian model probability densities—practices and algorithms. Pattern Recogn. 39, 1346–1358 10.1016/j.patcog.2006.01.005

[B53] Paek A. Y. Agashe H. A. Contreras-Vidal J. L. (2014). Decoding repetitive finger movements with brain activity acquired via non-invasive electroencephalography. Front. Neuroeng. 7:3. 10.3389/fneng.2014.00003 24659964 PMC3952032

[B54] Peterson D. A. Knight J. N. Kirby M. J. Anderson C. W. Thaut M. H. (2005). Feature selection and blind source separation in an EEG-based brain-computer interface. J. Appl. Sig. Proc. 19, 3128–3140 10.1155/ASP.2005.3128

[B55] Pfurtscheller G. Brunner C. Schlögl A. Lopes da Silva F. H. (2006). Mu rhythm (de)synchronization and EEG single-trial classification of different motor imagery tasks. Neuroimage 15, 153–159. 10.1016/j.neuroimage.2005.12.003 16443377

[B56] Pfurtscheller G. Kalcher J. Neuper C. Flotzinger D. Pregenzer M. (1996). On-line EEG classification during externally-paced hand movements using a neural network-based classifier. Electroenceph. Clin. Neurophysiol. 99, 416–425. 10.1016/S0013-4694(96)95689-8 9020800

[B57] Pfurtscheller G. Lopes da Silva F. H. (1999). Event-related EEG/MEG synchronization and desynchronization: basic principles. Clin. Neurophysiol. 110, 1842–1857. 10.1016/S1388-2457(99)00141-8 10576479

[B58] Pfurtscheller G. Neuper C. (2006). Future prospects of ERD/ERS in the context of brain-computer interface (BCI) developments. Prog. Brain Res. 159, 433–437. 10.1016/S0079-6123(06)59028-4 17071247

[B59] Prasad S. Bruce L. M. (2008). Limitations of principle component analysis for hyperspectral target recognition. IEEE Geosci. Remote Sens. Lett. 5, 625–629 10.1109/LGRS.2008.2001282

[B60] Presacco A. Forrester L. W. Contreras-Vidal J. L. (2012). Decoding intra-limb and inter-limb kinematics during treadmill walking from scalp electroencephalographic (EEG) signals. IEEE Trans. Neural Syst. Rehabil. Eng. 20, 212–219. 10.1109/TNSRE.2012.2188304 22438336 PMC3355189

[B61] Presacco A. Goodman R. Forrester L. Contreras-Vidal J. L. (2011). Neural decoding of treadmill walking from noninvasive electroencephalographic signals. J. Neurophysiol. 106, 1875–1887. 10.1152/jn.00104.2011 21768121 PMC3296428

[B62] Qin L. Ding L. He B. (2004). Motor imagery classification by means of source analysis for brain-computer interface applications. J. Neural Eng. 1, 135–141. 10.1088/1741-2560/1/3/002 15876632 PMC1945182

[B63] Quintero H. A. Farris R. J. Goldfarb M. (2011). Control and implementation of a powered lower limb orthosis to aid walking in paraplegic individuals, in Proceedings IEEE International Conference Rehabilitation Robotics (ICORR) (Zurich), 1–6. 10.1109/ICORR.2011.5975481 PMC3402219 22275679

[B64] Robinson N. Guan C. Vinod A. P. Ang K. K. Tee K. P. (2013). Multi-class EEG classification of voluntary hand movement directions. J. Neural Eng. 10:056018. 10.1088/1741-2560/10/5/056018 24018330

[B65] Saito K. Washimi Y. Takahashi A. Kaneoke Y. (1996). Slow negative potential preceding the onset of postural adjustment. Electroenceph. Clin. Neurophysiol. 98, 449–455. 10.1016/0013-4694(96)95004-X 8763504

[B66] Scheibe C. Ullsperger M. Sommer W. Heekeren H. R. (2010). Effects of parametrical and trial-to-trial variation in prior probability processing revealed by simultaneous electroencephalogram/functional magnetic resonance imaging. J. Neurosc. 30, 16709–16717 10.1523/JNEUROSCI.3949-09.2010 PMC6634862 21148010

[B67] Shibasaki H. Hallett M. (2006). What is the Bereitschaftspotential? Clin. Neurophysiol. 11, 2341–2356. 10.1016/j.clinph.2006.04.025 16876476

[B68] Siemionow V. Yue G. H. Ranganathan V. K. Liu J. Z. Sahgal V. (2000). Relationship between motor activity-related cortical potential and voluntary muscle activation. Exp. Brain Res. 133, 303–311. 10.1007/s002210000382 10958520

[B69] Slobounov S. Hallett M. Stanhope S. Shibasaki H. (2005). Role of cerebral cortex in human postural control: an EEG study. Clin. Neurophysiol. 116, 315–323. 10.1016/j.clinph.2004.09.007 15661110

[B70] Slobounov S. Johnston J. Chiang H. Ray W. (2002). Movement-related EEG potentials are force or end-effector dependent: evidence from a multi-finger experiment. Clin. Neurophysiol. 113, 1125–1135. 10.1016/S1388-2457(02)00123-2 12088709

[B71] Su T. Dy J. G. (2007). In search of deterministic methods for initializing k-means and Gaussian mixture clustering. Intell. Data Anal. 11, 319–338.

[B72] Sugiyama M. (2007). Dimensionality reduction of multimodal labeled data by local Fisher discriminant analysis. J. Mach. Learn. Res. 8, 1027–1061.

[B73] Velu P. D. de Sa V. R. (2013). Single-trial classification of gait and point movement preparation from human EEG. Front. Neurosci. 7:84. 10.3389/fnins.2013.00084 23781166 PMC3678086

[B74] Vlassis N. Likas A. (2002). A greedy EM algorithm for Gaussian mixture learning. Neural Proc. Lett. 15, 77–87 10.1023/A:1013844811137

[B75] Vuckovic A. Sepulveda F. (2008). Delta band contribution in cue based single trial classification of real and imaginary wrist movements. Med. Biol. Eng. Comput. 46, 529–539. 10.1007/s11517-008-0345-8 18418635

[B76] Wagner J. Solis-Escalante T. Grieshofer P. Neuper C. Muller-Putz G. Sherer R. (2012). Level of participation in robotic-assisted treadmill walking modulates midline sensorimotor EEG rhythms in able-bodied subjects. Neuroimage 63, 1203–1211. 10.1016/j.neuroimage.2012.08.019 22906791

[B77] Waldert S. Preissl H. Demandt E. Braun C. Birbaumer N. Aertsen A.. (2008). Hand movement direction decoded from MEG and EEG. J. Neurosci. 28, 1000–1008. 10.1523/JNEUROSCI.5171-07.2008 18216207 PMC6671004

[B78] Walter W. G. Cooper R. Aldridge V. J. McCallum W. C. Winter A. L. (1964). Contingent negative variation: an electric sign of sensorimotor association and expectancy in the human brain. Nature 203, 380–384. 10.1038/203380a0 14197376

[B79] Winchester P. McColl R. Querry R. Foreman N. Mosby J. Tansey K.. (2005). Changes in supraspinal activation patterns following robotic locomotor therapy in motor-incomplete spinal cord injury. Neurorehabil. Neural Repair 19, 313–324. 10.1177/1545968305281515 16263963

[B80] Wolpaw J. R. Birbaumer N. McFarland D. J. Pfurtscheller G. Vaughan T. M. (2002). Brain-computer interfaces for communication and control. Clin. Neurophysiol. 113, 767–791. 10.1016/S1388-2457(02)00057-3 12048038

[B81] Xu R. Jiang N. Lin C. Mrachacz-Kersting N. Dremstrup K. Farina D. (2014). Enhanced low-latency detection of motor intention from EEG for closed-loop brain computer interface applications. IEEE Trans. Biomed. Eng. 61, 288–296. 10.1109/TBME.2013.2294203 24448593

[B82] Zhang X. Liu Y. Zhang F. Ren J. Sun Y. L. Yang Q. (2012). On design and implementation of neural-machine interface for artificial legs. IEEE Trans. Ind. Informatics 8, 418–429 10.1109/TII.2011.2166770 PMC3290414 22389637

[B83] Zhong Z. Dong M. Bai-kun W. Long-long C. (2007). Event-related EEG-changes during attempted standing up task, in Joint Meeting 6th International Symposium Noninvasive Functional Source Imaging of the Brain and Heart & International Conference Functional Biomedical Imaging (NFSI-ICFBI) (Hangzhou), 66–69.

